# Kinetic and Isotherm Studies of Organic and Inorganic Anions Adsorption from Water by Quaternized Pentablock Copolymeric Film (PTBr)

**DOI:** 10.3390/polym17121624

**Published:** 2025-06-11

**Authors:** Simona Crispi, Simona Filice, Viviana Scuderi, Massimo Zimbone, Daniela Iannazzo, Consuelo Celesti, Silvia Scalese

**Affiliations:** 1 Istituto per la Microelettronica e Microsistemi—Consiglio Nazionale delle Ricerche (IMM-CNR), Ottava Strada n.5, 95121 Catania, Italy; simona.crispi@cnr.it (S.C.); viviana.scuderi@imm.cnr.it (V.S.); silvia.scalese@imm.cnr.it (S.S.); 2 Istituto per la Microelettronica e Microsistemi—Consiglio Nazionale delle Ricerche (IMM-CNR), Via S. Sofia 64, 95123 Catania, Italy; massimo.zimbone@cnr.it; 3 Department of Engineering, University of Messina, Contrada Di Dio, 98166 Messina, Italy; daniela.iannazzo@unime.it (D.I.); consuelo.celesti@unime.it (C.C.)

**Keywords:** pentablock copolymer, adsorption, nitrate ions, methyl orange, water treatment

## Abstract

Nowadays, nitrate ions and azo dyes are a significant source of water pollution due to their high toxicity, persistence, and potential to be carcinogenic. Both contaminants are the result of anthropogenic sources, such as sewage or industrial wastewater discharge; the first one results also as a consequence of the intensive use of fertilizers. In this work we report the use of a new quaternized pentablock copolymer (PTBr) for the removal of nitrate ions and methyl orange (MO) dye from water by adsorption processes. Morphological, chemical, and thermal properties of the pentablock copolymer were investigated, respectively, by scanning electron microscopy (SEM), Attenuated Total Reflectance Infrared Spectroscopy (ATR-FTIR) (FT-IR), and X-ray photoelectron spectroscopy (XPS), thermal gravimetric analysis (TGA), and differential scanning calorimetry (DSC) analyses. Anionic removal ability and adsorption rate in water solutions containing either a single contaminant species or a mix of the two contaminants were studied by UV–VIS absorbance spectroscopy as a function of time and initial concentration. The presence of imidazole groups confers on PTBr a positive charge and a hydrophilic character that are responsible for an effective removal of anions from water. PTBr film reports an adsorption efficiency of 10.15 mg/g for nitrate removal and this value is in line with others reported in the literature. In the case of the simultaneous presence of nitrate and MO, it is found that nitrate ions removal is slightly affected by the presence of the dye, since both contaminants compete for electrostatic interaction with imidazole groups. On the contrary, the dye removal does not show significant change with or without the presence of nitrate ions, probably due to other kinds of interaction that it can establish with the polymer surface (π-π interaction). The adsorption process and the related mechanisms are described using kinetic and isothermal models. Despite a certain reduction in the adsorption efficiency for one of the investigated contaminants, the results confirm the possibility of using the quaternized pentablock copolymer for the co-adsorption of both inorganic and organic anions.

## 1. Introduction

The world population is continuously growing and the latest world population projections indicate that it will reach 9 billion persons in 2037 and 10 billion persons in the year 2060 [1]. This implies an increase in food production and, generally speaking, water demand for the survival of living species; on the other hand, the increase in population has led to an increase in pollution by higher use of commercial products like pesticides, fertilizer, dyes, etc., by the increase in emissions and industrial activities. Consequently, more resources are urgently needed, including water resources, which already now seem to be not sufficient to satisfy the demand [2]. A larger availability of water can be achieved by removing contaminants from wastewater and reusing it for different purposes. Different treatment technologies have been developed to remove pollutants from water [3], depending on the type of contaminant to be removed. The main contaminants include organic molecules (dyes, antibiotics, etc.), pathogens (viruses, bacteria), or inorganic species (e.g., heavy metals, ions) [4]. Particular attention has been given to the removal of nitrate ions, whose sources are due to nitrogenous material and the formation of ammonia for natural water, while for wastewater the sources date back to the use of fertilizers, feed, and industrial treatments [5]. The nitrate ion represents the last stage of the nitrogen oxidation process; by leaching, it contaminates surface waters and aquifers, due to its solubility in water. It stimulates eutrophication and it is a toxic agent for humans; it enters the organism through the food chain, and it is reduced to nitrite causing abnormal increases in methemoglobin, i.e., a poor supply of oxygen to the tissues. In other cases, symptoms such as vomiting, diarrhea, and diabetes are observed [6]. The EPA (Environmental Protection Agency) sets limits to the nitrate concentration in water, specifically below 10 mg/L for drinking water intended for children, with a maximum of 45 mg/L for adults and 50 mg/L for water from aqueducts [7].

To reduce high content of nitrates from water, in most cases biological processes, reverse osmosis, electrodialysis or ion exchangers are used [8]. Biological processes are based on the development of specific biomass for denitrification [9]. This method is low-cost, efficient, and green, compared to other technologies used (reversed osmosis, ion exchangers, etc.); but it requires high operational time with the formation of organic sludge and the recovery of the biomass is not simple. Reverse osmosis is a physical process in which water is forced to pass through a semi-permeable membrane under pressure, retaining most of the dissolved materials [10]. Similarly, in the process of electrodialysis, ions are forced to migrate through selective semipermeable membranes under the effect of an electric field [11]. Electrodialysis is an efficient and flexible process but, due to the high costs involved, it is suitable for low water volumes and thus is not widely used in water treatment. Ion exchangers are granular, insoluble substances whose molecular structure contains basic or acid sites that can exchange negative or positive ions with the liquid they are put in contact with [12]. Technologies based on ion exchangers ensure process control and are easy to automate. The main disadvantages of this technology are due to the regeneration process of the resin and high costs.

Besides inorganic contaminants, common water contaminants include organic compounds like dyes, pesticides, pharmaceuticals, and surfactants. In particular, synthetic organic azo dyes are widely used in the textile, cosmetics, leather, pharmaceutical, paper, paint, and food industries [13]. Unfortunately, about 15–50% of azo-type textile dyes are released into wastewater during the dyeing process and affects the quality of water sources, with a bad impact on the environment and high risk for human health. Among the water-soluble azo dyes, methyl orange (MO) represents a carcinogenic water pollutant that can cause vomiting and diarrhea, and it can result in death at high levels of exposure. Furthermore, by-products generated from their degradation or metabolization are toxic [14]. As for nitrate ions removal, several methods have been investigated for organic dyes removal, such as reverse osmosis [15], electrodialysis [16], and photocatalytic [17] and photo-electrocatalytic [18] processes but, even though these methods are potentially efficient, they are expensive [19].

Adsorption represents a competitive method in the field of wastewater and air treatment [20] due to its characteristics of simplicity, flexibility, high efficiency, not generating secondary pollutants, and low cost [21]. The adsorption mechanism is determined by a mass transfer of the contaminant from water on the surface of an adsorbent (usually a powder) through chemisorption and physisorption phenomena [22]. A lot of recent research activity has been devoted to the study of new adsorbents with high efficiency, low cost, and reusability. Organic materials, such as resins [23,24] or more generally polymeric membranes [25], can be used as adsorption platforms for contaminants removal, with the advantage of low cost, easy recovery, and regeneration at the end of the process. Furthermore, the performance of these materials can be improved by developing nanocomposite polymeric films by embedding active fillers such as graphene oxide or inorganic semiconductors. Good results in contaminants adsorption and/or degradation were obtained, along with the possibility of easy recovery and reuse of the photocatalyst [26,27,28].

Another strategy to improve membrane performance is the use of copolymer structures characterized by polymeric block copolymers (BCPs), or structures capable of self-assembling, forming micelles and different architectures, such as lamellar, cylindrical, or cubic. New functionalities such as charge separation, selective adsorption, anti-fouling, and/or selectivity can be added by introducing chemical functionalization on the polymeric backbone [29]. As an example, the sulfonic acid group (acid) is commonly known to create ion exchange resins for the removal of organic and inorganic cationic contaminants [4,30,31,32], but also to gain new functionalities such as antibiofouling and antimicrobial properties [4,33]. For anions removal, positively charged basic groups are introduced; weakly basic anion exchangers possess a variety of amino groups such as mainly tertiary amino groups and, rarely, primary and secondary groups while strongly basic resins contain quaternary ammonium groups [34]. Recently, imidazolium-based ionic liquids are becoming increasingly popular in a variety of areas including separation science membranes and water purification agents [35]. The imidazole ring can be easily ionized by quaternization of the tertiary nitrogen atom resulting in a permanent positive charge. This charge makes imidazolium an active site for the removal of organic and inorganic anions such as negatively charged dyes [36] and nitrate ions [37]. Imidazole’s tunable structure, thermal stability (many ionic liquids do not exhibit degradation up to 275 °C), and relatively high ionic conductivity are other reasons to explain the high reported interest in the literature for this molecule. Another interesting property of imidazole ring is the high reactivity, with the ability to act as both proton donor and acceptor.

The 3-ring position easily undergoes S_N_2 reactions and similarly, the 1-position containing a secondary amine is a highly reactive site. In the presence of a strong base, the 2-position is deprotonated, allowing for chemical modification of the resulting carbene. Finally, molecular solubility and conductivity can be easily tuned by exchanging the mobile counter anion. This site also serves as a coordination point for transition metals; imidazole rings form coordination complexes in solution, which is a key aspect in many polymerization techniques used to synthesize polymers with narrow polydispersity [35].

In this work, we propose, for the first time, the use of a polymeric pentablock film, brominated and quaternized with methylimidazole (named PTBr) for the removal of MO and nitrate ions from wastewater. The film is characterized by a polymeric block copolymer structure that is thermally, mechanically, and chemically stable and the selective addition of imidazole groups confers on it a positive charge and a hydrophilic character, making it suitable for the selective removal of anions from water. Chemical, thermal, and physical properties of the polymer were fully investigated and the removal mechanism of the PTBr film was studied through adsorption kinetic models and adsorption isotherms. Indeed, in order to scale up adsorption to real-scale application, the appropriate study of kinetic adsorption and equilibrium conditions is absolutely necessary to predict the adsorption parameters as well as to quantitatively compare the adsorbent behavior for various experimental conditions. Finally, to simulate a real wastewater sample, the co-adsorption of nitrate ions and MO molecules by PTBr was studied.

## 2. Materials and Methods

### 2.1. Materials

**Figure 1:**
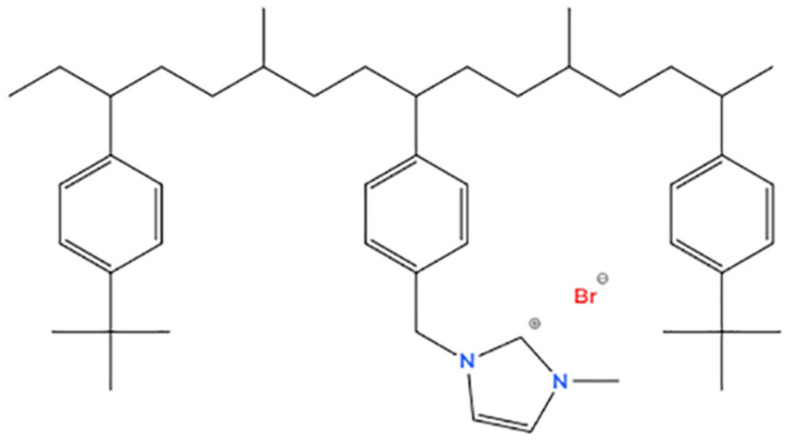
PTBr chemical structure.

A pentablock polymeric film named PTBr was provided by Kraton Polymers LLC. The ABCBA copolymer (t(BS)_15_-(EP)_12_-p(MS)_28_-(EP)_12_-t(BS)_15_) is composed of blocks of tert-butyl-styrene (tBS), ethylene-r-propylene (EP), and 4-methylstyrene (MS) in a molecular architecture that confers on the polymeric film mechanical resistance, flexibility, workability, and stability. The copolymer was brominated to approximately 61 mol% before it was quaternized with 1-methylimidazole to confer on it a hydrophilic character. The molecular structure of the block copolymer is reported in Figure 1. Milli-Q deionized water was used for the preparation of contaminant solutions and the polymeric film washing step. Potassium nitrate (KNO_3_ ≥ 99.0%) and Methyl Orange 0.1% solution (MO, indicator pH 3.10–4.4, red-yellow-orange) were purchased from Sigma-Aldrich and Merck, respectively.

### 2.2. Water Uptake Measurement

The water uptake of the PTBr membrane was verified according to the method described by Geise et al. [32,38] for another pentablock copolymer. In particular, the polymer membrane was immersed in Milli-Q water at room temperature for 48 h and quickly wiped with paper tissue in order to remove most of the free surface water before weighing. Afterwards, it was dried at room temperature and weighed again. The water uptake was calculated according to the following formula:

(1) $$Water\;Uptake\left(WU\right)=[(m_{wet}-m_{dry})/m_{dry}]\times 100$$

where $m_{wet\;}$ corresponds to the wet mass and ${\;m}_{dry}$ to the dry mass. The calculated value was 55% confirming that the introduction of imidazole groups on the polymer backbone confers on it a hydrophilic character.

### 2.3. Characterization Techniques

Morphology and thickness of the membrane were investigated by a field emission scanning electron microscope (Supra 35 FE-SEM by Zeiss, Oberkochen, Germany). In order to analyze the cross section of the membrane, a piece of membrane was dipped in liquid nitrogen and when it became rigid, it was easily broken in two parts.

Chemical composition and thermal properties were tested on the commercial film before and after a washing step: the membranes were soaked and washed in Milli-Q water at room temperature in order to remove eventual impurities, until the soaking solution stabilized at neutral pH measured by litmus paper, and to remove the bromide counterion leaving the imidazole groups free for adsorption. From now on the commercial sample will be indicated by the name PTBr, while the name w-PTBr indicates the sample after the washing step.

Chemical composition of the polymeric sample was investigated by Fourier Transform Infrared (FT-IR) Spectroscopy, using a Jasco FT-IR-4700 spectrophotometer equipped with an ATR (ATR-PRO ONE) with a diamond prism, and by X-ray photoelectron spectroscopy (XPS), using a PHI Genesis Multi-Technique Scanning XPS system, with a monochromatic Al Kα (1486.6 eV) X-ray beam and a 180° hemispherical electron energy analyzer. The Genesis system, equipped with a dual-beam charge neutralization system, allows turnkey neutralization of all types of insulating samples.

A PerkinElmer TGA 4000 instrument (PerkinElmer Inc., Waltham, MA, USA) was used for the thermogravimetric analysis. The scans were carried out under argon flow at a flow rate of 20 mL/min. Weighed amounts of PTBr and w-PTBr (5–10 mg) were poured into aluminum pan and subjected to heating from 100 °C to 1000 °C, at a heating rate of 10 °C min^−1^. Each sample was scanned in triplicate.

Differential scanning calorimetry (DSC) analyses were performed using a TA Q500 instrument (TA Instruments, New Castle, DE, USA) under nitrogen flow at a flow rate of 50 mL/min, from room temperature to 350 °C, with a heating rate of 5 °C/min.

The adsorption experiments were carried out by dipping 1 cm^2^ piece of w-PTBr membrane into 5 mL of KNO_3_ or MO or in KNO_3_ + MO mixed solutions and by measuring the changes in UV–Vis absorbance spectra with time. UV–Vis spectroscopic measurements were carried out using a Starline AvaSpec-2048L spectrometer equipped with an AvaLight-DH-S-BAL source (by Avantes) in a wavelength range between 200 and 800 nm, using wide optical window cuvettes (200–2500 nm). In particular, the contaminants adsorption was evaluated by means of the Lambert–Beer law, considering the absorbance peaks at 220 nm and 464 nm for nitrate ions and MO ions, respectively. The efficiency of adsorption was calculated for each solution by considering the ratio between the weight (mg) of adsorbed contaminant and the membrane weight (g) and was defined as Q_t_ (mg/g). The adsorption experiments were conducted at neutral pH and any variation of initial pH values for different contaminants solutions was monitored during adsorption experiments using litmus paper.

## 3. Results and Discussion

In this work, we propose, for the first time, the use of a polymeric pentablock film gaining imidazole groups (named PTBr) for the removal of MO and nitrate ions from wastewater. Before testing its removal efficiency, the film was washed in water to remove eventual impurities and bromide ions leaving the imidazole groups free for adsorption. For this reason, we report the characterizations of the film before and after the washing step. The aim of these characterizations is (i) to study the surface properties of PTBr film, (ii) to confirm the presence of free imidazole groups, and (iii) to confirm the removal of bromide ions from the film surface. All these aspects are essential for the adsorption processes. Furthermore, we investigate the thermal behavior of this film which is a key aspect for its application in real systems. Characterizations are conducted on both initial and washed film to be sure that the washing step does not affect the properties of PTBr film.

### 3.1. Characterization of Membranes

#### 3.1.1. Morphological Characterization

Film thickness was measured by SEM acquired on the cross section and the image is reported in Figure S1 of Supporting Information. The membrane is compact and homogeneous, with a thickness equal to 32.45 µm.

#### 3.1.2. Chemical Characterization: FT-IR and XPS

**Figure 2:**
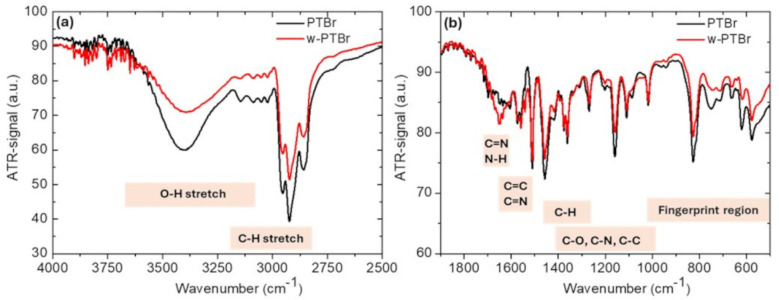
FT-IR spectra of a PTBr (black line) and w-PTBr (red line) in the range between (**a**) 4000–2500 cm^−1^, (**b**) 1900–500 cm^−1^.

For the PTBr (black line) and w-PTBr (red line) films, FT-IR spectra were acquired in the region between 4000 and 500 cm^−1^. In Figure 2a,b, the regions between 4000 and 2500 cm^−1^ and between 1900 and 500 cm^−1^ are shown, respectively.

In Figure 2a, PTBr and w-PTBr show a wide band centered on 3400 cm^−1^ related to the stretching vibrations of the hydroxyl group (OH group) due to the absorbed water. The peaks between 2800 cm^−1^ and 3200 cm^−1^ are correlated with the asymmetric and symmetric C-H stretching vibrations of the methyl groups. The region between 2400 cm^−1^ and 2000 cm^−1^ is not reported here, because it is strongly influenced by the presence of the environmental carbon dioxide signal. In Figure 2b, the region between 1700–1600 cm^−1^ is characteristic of C=N and N-H bonds. Both bonds are present in the polymer structure, and in particular, the increase in signal intensity for the w-PTBr sample could depend on the partial removal of Br atoms (due to the prolonged rinsing). In fact, considering the position of Br in the polymer chain (see Figure 1), its removal could favor the vibration of the bonds involving nitrogen. In the region between 1670–1500 cm^−1^ it is possible to observe the peaks related to the C=C and/or C=N double bond. C-H bonds are visible in the region between 1465–1365 cm^−1^. The region between 1400–1000 cm^−1^ is characterized by a whole series of peaks. Due to the structure of the polymer, they can be associated with both the C-N and C-C bonds. However, a contribution due to C-O bond is also possible if O is adsorbed from the environment, even if it is not contained initially in the original polymer chain.. At lower wavenumbers, from 1000 to 500 cm^−1^, the fingerprint region characteristic of each single molecule extends. This region is characterized by vibrations of the entire molecular skeleton.

The surface properties of PTBr film before and after the washing step were investigated by XPS analysis. Indeed, the adsorption process relies on surface properties of the adsorbent, in particular on the chemical and physical interactions with contaminants occurring on its surface. XPS survey spectra were acquired for PTBr and w-PTBr as reported in Figure S2 of the Supporting Information. The main peaks observed in the XPS spectra are C1s, Br3d, O1s, N1s, and Si2p. In particular, the presence of silicon is a contamination due to the membrane process preparation with a total amount below 2%. The peak area ratios of each element with respect to carbon are reported in Table S1 of Supporting Information for samples before and after the washing treatment. In agreement with FT-IR analysis, the washing treatment led to the partial removal of bromine ions, as evidenced by the reduction in Br/C from 0.205 to 0.0195 for PTBr and w-PTBr, respectively. Similarly, if we compare the Br/N ratio for both samples, this value reduced from 1.405 to 0.207 after the washing step.

**Figure 3:**
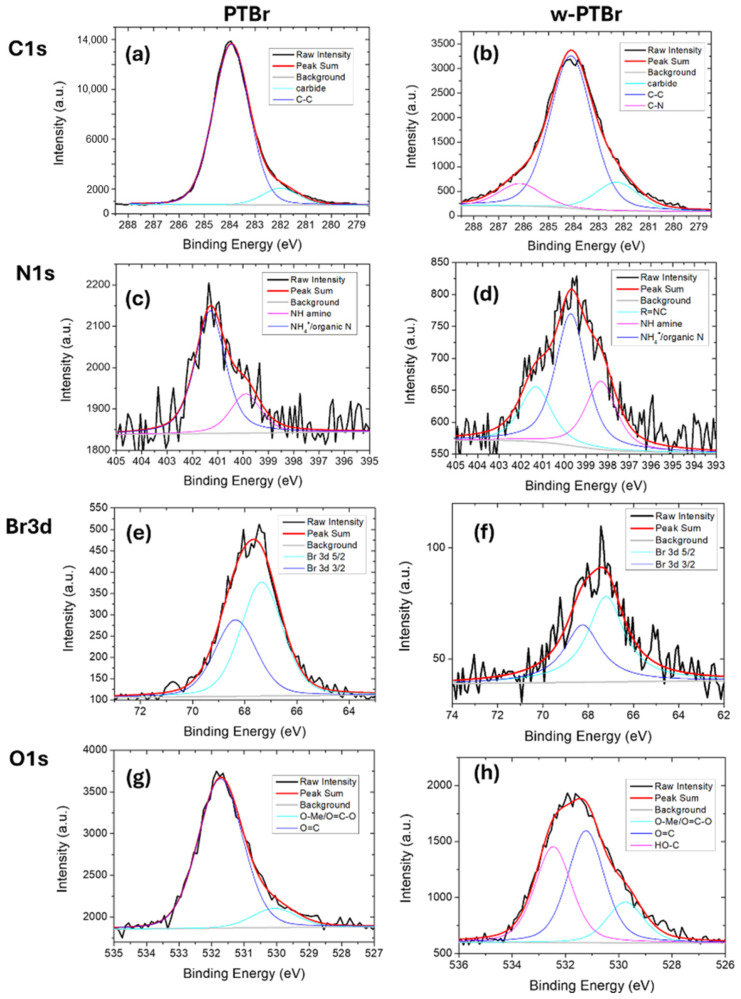
XPS spectra of PTBr and w-PTBr samples for (**a**,**b**) C1s, (**c**,**d**) N1s, (**e**,**f**) Br3d, and (**g**,**h**) O1s. Black continuous lines indicate the acquired spectra, red lines are the fits of the spectra, grey lines are the subtracted baseline, and the other colored lines are the components obtained by deconvolution of each peak.

Figure S3 of the Supporting Information reports the XPS spectra acquired on PTBr and w-PTBr films related to carbon, nitrogen, oxygen, and bromine. All spectra were shifted to align the C1s peak to 284 eV. Table 1 reports the relative contributions (%) for each element peak calculated by deconvolution of each element spectra reported in Figure 3. 

The C1s spectrum of commercial PTBr film is deconvoluted into two peaks at 282.0 and 283.9 eV that are associated to carbide or CH or C vacancies and C-C bonds, respectively. We suppose that the first peak is due to the synthetic procedure of polymeric backbone. Besides these two peaks, for w-PTBr, a new peak is observed at 286.15 eV related to C-N bonds of imidazole groups. Similarly, for the N1s XPS peak we calculated two contributions for PTBr itself at 401.3 eV and 399.9 eV related to NH_4_^+^ and NH of amine, respectively, while the same peak for w-PTBr is deconvoluted into three contributions related to NH_4_^+^ (401.2 eV), NH of amine (399.7 eV), and R=N-C (398.3 eV). All these contributions are related to imidazole functionalization. As previously evidenced by IR analysis and the Br/N and Br/C ratios calculated by elemental analysis, contributions of C and N elements of imidazole are more evident after the washing step as a consequence of the partial removal of bromine.

For both PTBr and w-PTBr, the Br3d peak is found at 68 eV and can be deconvoluted into Br3d_5/2_ and Br3d_3/2_ contributions, due to spin–orbit coupling, distant ~1.04 eV in energy and with an intensity ratio of 0.671.

Oxygen is present on PTBr film surface as contamination; two peaks related to O=C-O and O=C are observed at 530.1 and 531.7 eV, respectively. For w-PTBr film, besides the O=C-O and O=C contributions observed at 529.7 and 531.2 eV, respectively, another peak at 532.5 eV is observed due to HO-C originating from the washing step.

#### 3.1.3. Thermal Analysis

The thermal behavior of both the PTBr membrane and w-PTBr was evaluated using Differential Scanning Calorimetry (DSC) and Thermogravimetric Analysis (TGA).

As reported, the treatment with water of imidazole salts with bromide as the counterion can induce changes in the structural, thermal, and chemical properties of the bound structure after dissociation [39]. Moreover, the imidazole cation may interact with water, forming hydrogen bonds with the nitrogen or bromide anion, potentially altering ion pairing and charge distribution within the molecule [40].

**Figure 4:**
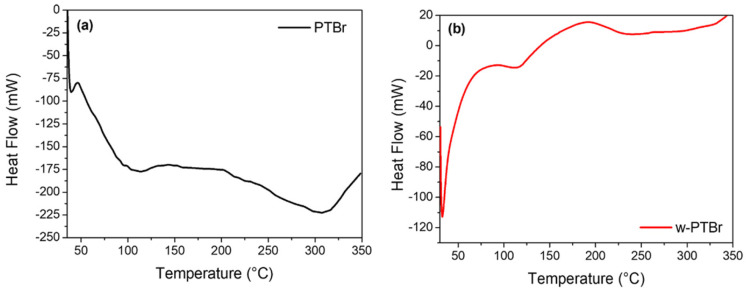
DSC thermograms of the PTBr films (**a**) as received and (**b**) after washing. The experiments were performed under N_2_ atmosphere.

These structural changes are clearly reflected in the DSC thermograms of both the water-treated and the original membrane (Figure 4).

For both samples, a decrease in heat flux is observed at 32 °C, indicating an endothermic transition, likely corresponding to the glass transition temperature (T_g_) of the polymer structure, where the material undergoes a reduction in molecular mobility. For the PTBr membrane, a decrease in heat flux was observed between 100 °C and 124 °C, indicating another endothermic peak, suggesting a phase transition or the beginning of thermal degradation of the ionic material. For the w-PTBr membrane, the same decrease in heat flux was recorded between 76 °C and 137 °C, indicating an endothermic process, probably due to changes in thermal stability or the breakdown of the ion network after washing. The observed enlargement and shift of this endothermic peak with respect to the starting sample suggest a change in the thermal properties of the polymeric material after washing, indicating alterations in the intermolecular interactions and a lower degree of crystallinity. Furthermore, w-PTBr shows an increase in heat flux between 167 °C and 210 °C, indicating an exothermic peak, that corresponds to the crystallization or solidification of hydrophobic groups (such as alkyl side chains) after washing. Finally, for the PTBr membrane, a decrease in heat flow between 257 °C and 337 °C was recorded, representing another endothermic peak, likely due to the decomposition of the membrane material. These results indicate that the washing process alters the thermal behavior of the membrane, likely by disrupting ionic interactions and maybe promoting the crystallization of hydrophobic components. In summary, the results of DSC analyses clearly demonstrate how the washing process in these imidazole salt-based membranes leads to changes in crystalline structure and thermal transitions due to shifts in molecular arrangement and interactions as also reported for other similar systems [41].

**Figure 5:**
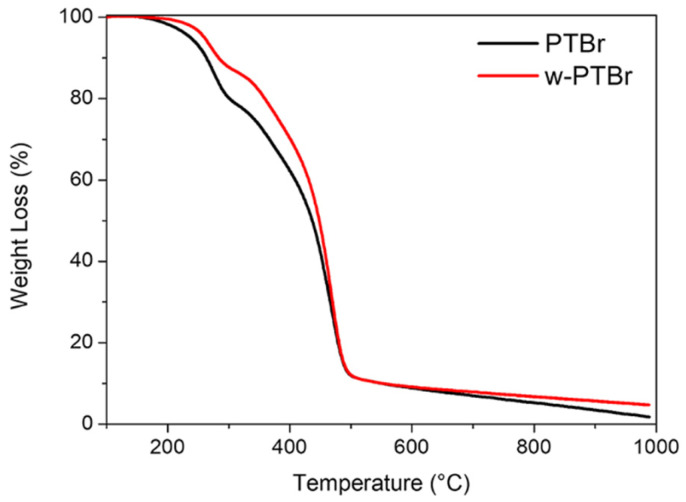
TGA profiles of PTBr films as received and after washing. The experiments were performed under Ar atmosphere.

The TGA curves of both samples, recorded under an inert atmosphere, show three distinct regions of mass loss (Figure 5). First, a slight weight reduction of approximately 10% occurs at temperatures below 150 °C, corresponding to the loss of physically bound water from the membrane.

The second mass loss, observed between 260 °C and 400 °C, can be attributed to the loss of imidazole groups, as also reported in the literature for similar systems [42,43].

The final weight loss, above 400 °C, is linked to the thermal degradation of the polymer backbone. Similar to the DSC analyses, the difference in weight loss can be attributed to the dissociation of the imidazole salt following water treatment. The water-treated membrane exhibits improved thermal stability, likely due to the formation of hydrogen bonds, which may reduce the rate or extent of membrane decomposition [44].

In conclusion, chemical and thermal analysis confirmed that bromide removal occurred after washing in Milli-Q water; as a result, the imidazole groups are thus positively charged, promoting both the formation of hydrogen bonds that improve membrane thermal stability, and also serving as active sites for anions adsorption. A pretreatment of the membrane with water not only modifies the polymeric structure, improving its properties, but also prevents the exchange of bromide ions with the surrounding environment, thereby avoiding the release of secondary products as can occur by using some exchange resins [45].

### 3.2. Adsorption Experiments—Nitrate Ions

**Figure 6:**
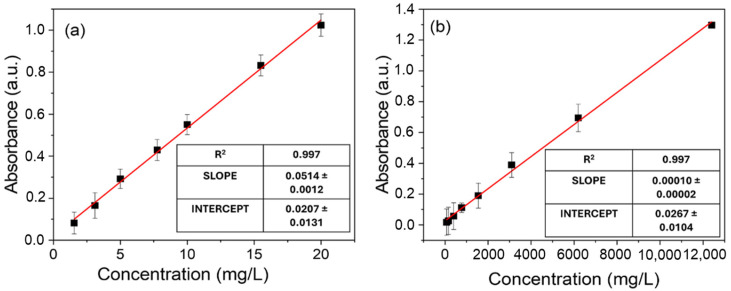
Calibration curves for two NO_3_^−^ concentration ranges: (**a**) from 1.55 to 20 mg/L considering the absorbance peak at 220 nm; (**b**) from 775 to 12,400 mg/L considering the absorbance peak at 300 nm. Black dots are the experimental data and red lines indicate linear fitting. Relative errors for each point are reported (black lines).

Nitrate ion adsorption experiments were performed as reported in Section 2.3. Before tests, an appropriate calibration curve from low to high concentrations of nitrate ions was performed to verify the linearity of the Lambert–Beer law within the investigated concentration range, i.e., from 1.55 mg/L to 12,400 mg/L. UV–Visible absorbance spectra of nitrate solutions at different concentrations are reported in Figure S4 of the Supporting Information and, as expected [46], two absorbance peaks are observed at 220 nm and 300 nm, respectively. These peaks can be used for calibration in two NO_3_^−^ concentration ranges: in Figure 6a,b we report, respectively, the calibration curves for lower concentration values (from 1.55 to 20 mg/L), obtained by following the absorbance peak at 220 nm, and for higher concentration values (from 775 to 12,400 mg/L), considering the peak at 300 nm. The use of two different peaks in the two ranges of concentrations allows to obtain a linear dependence of the absorbance on the concentration values.

The adsorption experiments as a function of time were performed at different initial nitrate concentrations in the range 0–15.50 mg/L. The UV–Visible absorbance spectra of nitrate solutions with initial NO_3_^−^ concentration of 1.55 mg/L, 3.10 mg/L, 7.75 mg/L, and 15.50 mg/L in the presence of a w-PTBr membrane are reported in Figure S5 of Supporting Information.

**Figure 7:**
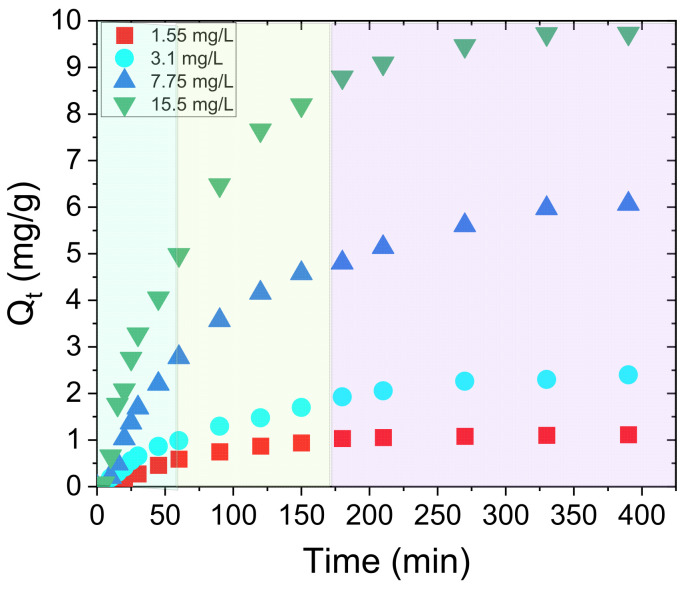
Q_t_ values calculated as a function of time for different initial NO_3_^−^ concentrations. The three different colored areas indicate three different regions with three different slopes.

In this lower concentration range, the evolution of the 220 nm absorbance peak in the presence of the w-PTBr film was followed versus time, according to the calibration curve shown in Figure 6a. For all the investigated solutions, no changes in the shapes of the UV–Visible spectra were observed over time after immersing the polymeric film. Similarly, the absorbance peak at 220 nm decreased with time confirming the nitrate removal ability of w-PTBr film by electrostatic interaction between nitrate ions and positively charged imidazole groups on the polymer backbone. Furthermore, the removal efficiency was estimated as the amount of ions adsorbed by mass unit of w-PTBr sample for each solution, i.e., Q_t_, (mg/g). Figure 7 reports the Q_t_ values versus adsorption time for each solution.

For all curves, the Q_t_ values increased with time until reaching a constant final value of 0.93, 2.51, 6.10, and 10.15 mg/g, respectively, for increasing nitrate initial concentrations. The higher value is observed, as expected, for the highest NO_3_^−^ concentration (15.50 mg/L). In this case, a higher mass transfer from the solution to the film surface is induced as a consequence of the higher concentration gradient at the liquid/membrane interface [47]. In each curve of Figure 7, three different regions according to three different slopes can be identified (evidenced by three different colored areas): the highest slope value is observed in the first hour; this value decreases for the following two hours until a plateau is reached in the last hour. This outlines a change in the kinetic constant of the adsorption process over time or the copresence of different adsorption mechanisms.

If we consider that the adsorption mainly occurs on imidazole active sites that are the 61 %mol of total polymer moles, all the Q_t_ values reported are underestimated. Therefore, all reported Q_t_ values should be rescaled considering the moles of active sites.

Table 2 below compares the Q_t_ values calculated as (i) the ratio between adsorbed NO_3_^−^ (mg) and w-PTBr moles or (ii) adsorbed NO_3_^−^ (mg) and moles of imidazole sites.

If one consider the moles of the active groups (imidazole) instead of the entire polymer, the calculated Qt values would be higher, as shown in Table 2. However, in the following paragraphs we will refer to Q_t_ values reported in Figure 7, calculated as mg/g, since we will compare the nitrate adsorption process on w-PTBr with the adsorption of another kind of contaminant (i.e., MO) occurring on different sites of the polymeric backbone and not only on imidazole groups. Similarly, in conclusion we will compare our results with the literature results obtained for different kinds of adsorbents, where the total adsorbent mass is reported.

To deeply investigate the observed trends and adsorption mechanisms occurring, we report below a detailed kinetic analysis following three main kinetic models, i.e., the pseudo-first-order (PFO), the pseudo-second-order (PSO), and the intraparticle diffusion models.

The adsorption process occurs in three main steps: an initial mass transfer of the solute from the external solution to the adsorbent surface, followed by diffusion of the adsorbate to the adsorption sites, and finally, the adsorption and desorption of solute molecules on and from the sorbent surface. For the most widely used models, the last step, in which the surface reactions occur, mainly controls the overall rate of the sorption process. The surface reaction involves both physical interactions (Van der Waals forces) and chemical interactions (functional group coordination, ion exchange, and redox) based on the selected adsorbent. Indeed, kinetics are influenced by the surface complexity of the adsorbent and by the solute concentration [47]. The PFO and PSO models rely on the above assumptions and their main advantage is simplicity to manage experimental data with no need to use advanced computational procedures.

At the end of 19th century, Lagergren presented the pseudo-first-order equation describing the rate of sorption in liquid-phase systems [48]. The overall sorption process is controlled by the rate of adsorption/desorption processes seen in terms of a chemical reaction on the adsorbent surface. The linear expression of the PFO model is reported in Equation (2):

(2) $$\ln\left(Q_{e}-Q_{t}\right)=lnQ_{e}-k_{1}t$$

where k_1_ is the rate constant (min^−1^), and Q_t_ and Q_e_ are the amount of adsorbate (mg) adsorbed for mass unit of adsorbent (g) at time t or at equilibrium, respectively. The value of k_1_ is determined by plotting ln (Q_e_ − Q_t_) versus t. Higher k_1_ values mean shorter times required to complete the adsorption process. Many experimental studies have confirmed that the value of the k_1_ parameter can be both dependent on and independent of the applied operating conditions [47].

In the pseudo-second-order kinetic, the ion exchange reactions occur on adsorbent surface determining the total removal kinetics. These reactions have a kinetic order of two with respect to the number of adsorption sites available for the exchange [49].

The PSO equation is reported in Equation (3):

(3) $$t/Q_{t}=1/(k_{2}{Q_{e}}^{2})+(t/Q_{e})$$

where Q_t_ and Q_e_ are the amount of adsorbate adsorbed onto the adsorbent (mg/g) at time t and at equilibrium, respectively, and k_2_ is the rate constant per min (g mg^−1^min^−1^). The Q_e_ and k_2_ parameters can be determined directly from the slope and the intercept of the t/Q_t_ against t plot. The value of kinetic constant strongly depends on the applied operating conditions, i.e., initial solute concentration, pH, temperature, etc. In particular, the k_2_ value decreases with the increasing initial contaminant concentration since higher adsorption times are required [47,50,51,52,53,54,55].

This model has been widely applied to describe chemisorption involving covalent forces and ion exchange between the adsorbent and adsorbate [56]. With respect to the PFO model, the PSO one has the advantage of directly calculating equilibrium capacity Q_e_ [57]. Most of the adsorption studies reported a more reliable description of real adsorption processes by PSO than by PFO. This is mainly attributed to the fact that the PSO model allows to estimate the Q_t_ value of equilibrium. Indeed, the accuracy of the PFO model largely decreases for data in the equilibrium range since Q_e_ and Q_t_ are almost the same and ln (Q_e_ − Q_t_) becomes abnormally large. Anyway, considering only the PSO model to describe adsorption process may lead to the conclusion that the rate determining step of adsorption is chemisorption. This can be misleading, and the experimental process should also be checked using diffusion-based models.

Diffusion models, on the other hand, assume diffusion as the rate-limiting factor and are divided into external mass transfer model and internal diffusion model [58]. In the former, the mass transport of the adsorbate to the adsorbent is the rate limiting step. However, the effect of transport from the bulk solution to the sorbent surface can be eliminated by solution stirring. The internal diffusion model takes into account the mass transfer into the interior of the particle as the slowest step. Equation (4), as described by Weber and Morris [57], may be applied in the determination of the intraparticle diffusion rate constant, K, and the boundary resistance, C:

(4) $$Q_{t}=K(t^{0.5})+C$$

where Q_t_ (mg/g) is the amount of adsorbate adsorbed onto the adsorbent at time t, K is the rate constant (mg/g) min^0.5^, and C determines the boundary layer effect (i.e., higher values, larger film diffusion resistance) and is linked to external mass transfer [47]. K and C are estimated by the plotting of Q_t_ versus t^0.5^. When C = 0, the intraparticle diffusion alone is the rate limiting step; when C > 0, the film diffusion also takes place. The larger the C value, the greater is the boundary layer effect, i.e., the film resistance to mass transfer surrounding the adsorbent, and this increases with the adsorption rate of ions on the adsorbent surface [30].

To study adsorption kinetics, kinetic Equations (2)–(4) have been applied, and the suitability of any model depends on the error level, i.e., correlation coefficient (R^2^) or Sum of Squared Errors (SSE). The model that has the R^2^ closest to 1 is the one that better describes the mechanism of the removal process.

Figure S6 of Supporting Information reports the linear fitting of the PFO, PSO, and diffusion models according to different nitrate initial concentrations, considering the adsorption experiments of nitrate ions at different initial concentrations by 1 cm^2^ of w-PTBr film (see Figure 7 and Figure S5 of Supporting Information). The fitting parameters are reported in Table S2 of Supporting Information. According to the R^2^ values, the kinetic model that best fits experimental data is the PFO, independently of the initial nitrate concentrations. This outlines that the overall sorption process is controlled by the rate of nitrate surface reactions occurring on the w-PTBr surface, i.e., by the electrostatic interaction between nitrate ions and imidazole active sites.

Table 3 reports the coefficient R^2^, the kinetic constant k_1_, and Q_e_ values estimated by PFO linear fitting parameters for all investigated nitrate concentrations. Estimated Q_e_ values are compared with the experimental Q_e_ values after 24 h of adsorption when the absorbance spectra of nitrate solutions with w-PTBr films did not change any more. K_1_ values increase as a function of concentration, and it is almost the same for the last two concentrations. Q_e_ values increase with initial nitrate concentrations as also the error, considering the estimated value with respect to experimental ones, increases with increasing nitrate concentration. Indeed, at higher initial concentrations the accuracy of this model to estimate k_1_ and Q_e_ decreases, as explained above.

As shown in Figure 7, independently of the initial nitrate concentrations, three different adsorption steps according to three different slopes can be identified. Therefore, the PSO and diffusion intraparticle models also have to be considered.

Table S3 of the Supporting Information reports the fitting parameters of the PFO, PSO, and diffusion kinetic models for nitrate adsorption at 1.55, 3.10, 7.75, and 15.50 mg/L initial concentrations in three different time ranges, i.e., for the first hour, from the first to the third hour, and for the last time range (up to 330 min). The linear fittings of experimental data according to the three models for each initial nitrate concentration are reported in Figures S7–S10, respectively, of the Supporting Information.

For all the solutions investigated, the mechanisms described by the PFO and intraparticle diffusion models are predominant and quite similar in the first hour, while the PSO model starts to be more valid from the second hour of the experiment. Initially, nitrate ions are adsorbed on the film surface by direct interaction with imidazole groups. After the first hour, the active sites start to be saturated and different surface reactions can take place on the film surface. This is suggested by the possibility of fitting the experimental data with the PSO model, which has been widely applied to describe chemisorption involving covalent forces and ion exchange between the adsorbent and adsorbate [47]. Simultaneously, the depletion of nitrate ions by surface interaction on the polymeric film increases the concentration gradient at the solid/liquid interface, favoring ion diffusion, and this is in agreement with the possibility of using the intraparticle diffusion model to explain the process.

To sum up, the adsorption process is mainly determined by the surface interactions (both chemical and physical) between nitrate ions and active sites on the polymeric film, and the nature of these interactions evolves with adsorption time. Ion diffusion also plays a crucial role in all the investigated ranges as a direct consequence of ions adsorption on polymeric surface.

Besides kinetic parameters, it is fundamental to obtain information at equilibrium conditions in order to gain a complete understanding of adsorption phenomena.

By graphing the amount of adsorbate for adsorbent unit with respect to adsorbate concentration, an adsorption isotherm curve can be obtained. Based on the shape of these curves, adsorption isotherms can be categorized into different types, each describing a specific interaction mechanism between pollutants and adsorbent materials [59].

Different isotherms are used which are distinguished in two-parameter isotherms (such as Freundlich, Langmuir), three-parameter isotherms (such as Redlich–Peterson, Sips, Toth, and so on) or four-parameter isotherms (Fritz–Schlunder, Bauder, and so on). Among these, the Langmuir and Freundlich are the most used by researchers as they allow to determine whether it is a homogeneous adsorption, in the case of the Langmuir, or heterogeneous, in the case of the Freundlich [60].

The Langmuir isotherm model relies on the assumption that rates of adsorption and desorption should be equal, adsorbate–adsorbent interactions are negligible, and the thickness of the adsorbed layer is one molecule (monolayer adsorption). In other words, the Langmuir isotherm model assumes that adsorption is homogenous, i.e., all active sites should have equal affinity towards the adsorbate that is blocked on the adsorbent surface (no transmigration in the surface plane occurs) [61,62,63].

The linear expression of the Langmuir model is given by Equation (5):

(5) $$1/Q_{e}=1/{(K}_{L}⨯Q_{max\;})⨯1/C_{e}+1/Q_{max\;}$$

where *C_e_* is the adsorbate concentration in the solution at equilibrium (mg/L), *Q_e_* the solute mass adsorbed per unit adsorbent mass at equilibrium (mg/g), *K_L_* the constant of the Langmuir isotherm (L/mg), and *Q_max_* relates to the maximum adsorption capacity (mg/g). The *Q_max_* and *K_L_* constants are calculated from the slope and intercept of the linear fitting of Equation (5). These parameters give information on the solute affinity to the adsorbent. The Freundlich adsorption isotherm model describes the reversible and non-ideal adsorption process, applied to the multilayer adsorption. The Freundlich isotherm model expression defines the heterogeneity of the surface as well as the distribution of the active sites.

The linear Freundlich isotherm may be written as reported in Equation (6):

(6) $$logQ_{e}=\log C_{e}+logK_{F}$$

where *C_e_* is the adsorbate concentration in the solution at equilibrium (mg/L), *Q_e_* the solute mass adsorbed per adsorbent mass unit at equilibrium (mg/g). $K_{F}$ and 1/*n* can be determined from the linear plot of $logQ_{e}$ versus $\log C_{e}$, respectively. $K_{F}$ is the constant of the Freundlich isotherm (L^1/*n*^ mg^(1−1/*n*)^/g), and 1/*n* is the Freundlich exponent. Higher $K_{F}$ values indicate higher affinity for adsorbate; 1/*n* values indicate if adsorption is favored (0.1 < 1/*n* < 1), unfavored (1/*n* > 1), or irreversible (1/*n* = 1).

Figure S11 of Supporting Information reports the linear fits of equilibrium data for nitrate adsorption on w-PTBr films according to both the Freundlich and Langmuir models. The fitting parameters are reported in Table S5 of Supporting Information. Even if high R^2^ values are obtained for both models, the adsorption parameters extrapolated by the linear fit have no physical sense. Indeed, a negative slope is obtained for the Langmuir fitting indicating a negative value of Q_max_ that has no sense. By using the Freundlich model, a value of 1/*n* equal to 1.7 is obtained, indicating an unfavorable process. These results underline that the investigated models are not suitable to explain the adsorption process of nitrate ions on w-PTBr films.

The Freundlich and Langmuir isotherm models have been developed for gas adsorption on solids and do not consider the solvent–adsorbent interaction that is a key aspect in processes like ours [64].

In the case of the adsorption of solid solutes from a solution onto a solid, we can consider Giles isotherm classification (C, H, L, and S) [65]. In this case, Φ, calculated as the ratio between the amount of adsorbed contaminant at equilibrium with respect to the moles of active sites, is plotted versus solute concentrations and a typical S-type adsorption isotherm is observable. The sigmoidal shape indicates the presence of a transition point resulting from the competition between the solute and the solvent for binding sites on the adsorbent, in our case between nitrate ions and water, respectively.

**Figure 8:**
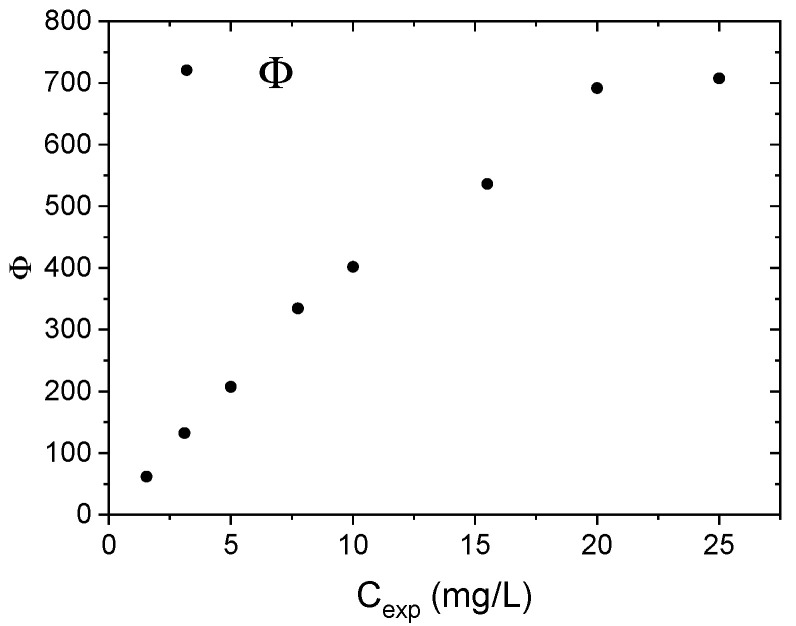
The ratio between the amount of adsorbed nitrate with respect to the moles of active sites (imidazole) versus nitrate concentrations.

In an S-type isotherm it is possible to identify (i) a first region indicating that water molecules adsorption is preferred with respect to solute adsorption; (ii) a second region, associated to an equilibrium condition between water molecules and solute, where the turning point can be identified; and (iii) the last region identified by a plateau, where solute adsorption overcomes the water molecules adsorption [66]. Figure 8 shows Φ, i.e., the ratio between the amount of adsorbed nitrate with respect to the moles of active sites (imidazole), versus nitrate concentrations. The amount of adsorbed nitrate is measured as the difference between the nitrate concentration at equilibrium and the initial one.

The above-reported plot confirms that the isotherm model valid for nitrate adsorption is more similar to an S-type than a linear one, even though the first region is missing; this means that the competition between water molecules and nitrate ions should be taken into account to describe our adsorption process, but solute adsorption is quite dominant over water adsorption within the investigated nitrate concentration range [65,67].

### 3.3. Adsorption Experiments—Organic Pollutants (MO Dye) Removal

**Figure 9:**
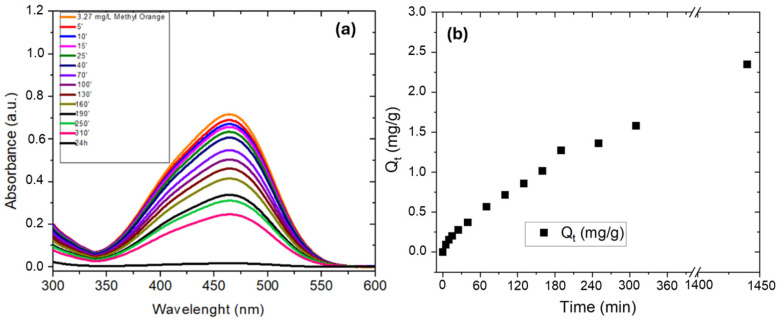
UV–Visible spectra of (**a**) MO solution (3.27 mg/L) where 1 cm^2^ of w-PTBr film was immersed and (**b**) Q_t_ values calculated as a function of time.

Figure 9a reports the UV–Visible spectra of MO solution (3.27 mg/L) where 1 cm^2^ of w-PTBr film was immersed. The Q_t_ values were estimated by the absorbance peak at 464 nm and their evolution with time is reported in Figure 9b.

The UV–Visible absorbance spectrum of MO dissolved in water at neutral pH shows a peak at 464 nm which is due to the azo linkage of MO [31] and was used to quantify the MO removal.

The absorbance peak at 464 nm decreases with time confirming that the w-PTBr membrane is able to adsorb MO molecules until their complete removal after 24 h. Similarly, Q_t_ values increase with dipping time up to a final value of 2.35 mg/g when all MO molecules are removed.

As for nitrate ions, we investigated the kinetics of the adsorption process using the three models i.e., PFO, PSO, and intraparticle diffusion.

Table 4 reports the kinetic parameters, i.e., the coefficient R^2^ and the kinetic constants k_1_, k_2_, and K for the PFO, PSO, and intraparticle diffusion models, respectively. Furthermore, we report the Q_e_ values estimated by the PFO and PSO models and the value C for the diffusion model.

All these parameters are estimated by the linear fitting of kinetic models shown in Figure S12 of Supporting Information and fitting parameters are reported in Table S5 of Supporting Information for all the kinetic models investigated.

As reported, the PFO model fits well the experimental data of MO adsorption with an estimated Q_e_ value of 2.29 mg/g, that is close to the experimental one calculated after 24 h of immersion (i.e., 2.35 mg/g). This means that the adsorption is mainly governed by surface reactions of MO molecules on the w-PTBr film surface, i.e., electrostatic interactions between negative dye molecules and positively charged imidazole groups and π-π interactions between aromatic rings of MO molecules and the polymer backbone.

### 3.4. Adsorption Experiments—Mixed Organic and Inorganic Pollutants Removal

**Figure 10:**
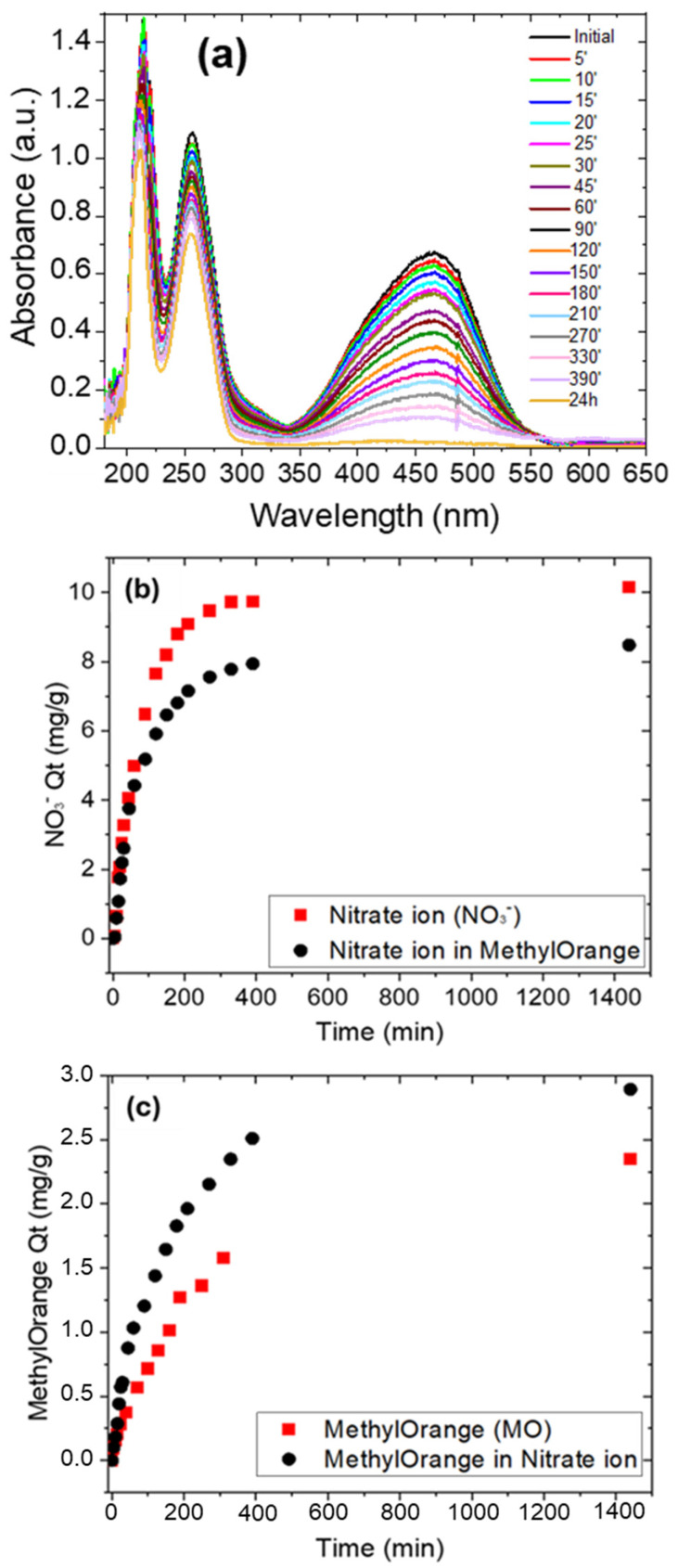
(**a**) UV–Visible spectra of MO solution (3.27 mg/L) mixed with KNO_3_ (15.50 mg/L) where 1 cm^2^ of w-PTBr film was immersed. (**b**) Q_t_ values calculated as a function of time for nitrate ions removal alone or in the presence of MO. (**c**) Q_t_ values calculated as a function of time for MO ions removal alone or in the presence of KNO_3_.

To simulate a wastewater sample containing organic and inorganic contaminants, a 5 mL aqueous solution containing both NO_3_^–^ (15.50 mg/L) and MO (3.27 mg/L) was prepared. The experiment was conducted following the methodology used for single species adsorption and following the peak at 220 nm for the nitrate ion and the peak at 464 nm for MO. Figure 10a reports the UV–Visible spectra of mixed solutions where w-PTBr film was immersed. To exclude any reciprocal interaction between the two contaminants affecting the shape of UV–Visible signals, the reference spectra of KNO_3_ and MO solutions, respectively, at the same initial concentrations are compared with the spectrum of mixed initial solutions. These are reported in Figure S13 of Supporting Information, where the spectral region between 200 and 300 nm is also shown in more detail, clearly showing the nitrate ions peak. The absorbance spectra evolution with time is reported in Figure 10a and the Q_t_ values for nitrate and MO removal were estimated by the absorbance peaks at 220 nm and 464 nm, respectively, as shown in Figure 10b,c. In the same figures a comparison between the mixed solution and single-contaminant solutions is reported.

The w-PTBr membrane was able to remove simultaneously the inorganic and organic molecules as indicated by the decrease in the absorbance peaks at 220 and 464 nm, respectively, in the UV–Visible spectra. However, due to a competitive adsorption effect, the Q_t_ values obtained after 24 h for the nitrate removal in mixed solution are slightly lower than in single-contaminant solutions. On the contrary, for MO removal the Q_t_ value after 24 h is quite similar for MO alone and in mixed solution. These values are reported in Table 5.

We can rationalize the observed differences considering that nitrate ions are adsorbed on PTBr film by ionic interaction with imidazole groups and this is reduced by the competitive presence of anionic MO molecules. On the contrary, MO can be adsorbed by both electrostatic and π-π interactions, and, therefore, since the adsorption efficiency is almost the same in the two cases, we deduce that even if the electrostatic interaction is reduced by the competitive presence of nitrate ions, the π-π interaction still occurs or is even enhanced.

As for single contaminant solutions, we report the kinetic analysis of adsorption process for nitrate and MO in mixed solutions.

In the Supporting Information, the linear fitting of kinetic models is reported in Figure S14, and the fitting parameters are reported in Table S6. With respect to single-contaminant adsorption experiments that follow the PFO model, the intraparticle diffusion model is the best to fit both MO and nitrate adsorption up to 330 min in mixed solution. When the two contaminants are both present in the solution, they compete in the adsorption process and the ability of each molecule to diffuse and reaching active sites is predominant over surface reactions.

This explanation is not exhaustive; even if the R^2^ values are close to 1, the fit is not good as observed in Figure S14. Indeed, observing the trend of Q_t_ values with time reported in both Figure 10b,c and Figure S14, different regions of linearity can be observed for both MO and nitrate molecules adsorption, suggesting that different mechanisms are involved. As done before for nitrate adsorption, we divide the adsorption experiments into three time ranges, i.e., the first hour, up to 180 min, and up to 330 min. Figures S15 and S16 and Table S7 of Supporting Information report the linear fitting of experimental data and relative fitting parameters according to the three models for both MO and nitrate adsorption.

For MO removal, the kinetic model that best fits experimental data in the first hour is intraparticle diffusion. After the first hour, the PFO and PSO models also become competitive and, in the last interval, all models have quite similar R^2^ values. This means that initially the key phenomenon is the diffusion of MO molecules on the film surface and then the surface reaction (both electrostatic and π-π interactions) starts to occur and becomes predominant. For nitrate ions adsorption, the diffusion model is predominant in the first hour within PFO, while the PSO is predominant for the remaining time. The kinetic parameters calculated for each model for MO and nitrate adsorption in mixed solution and different region times are reported in Table 6.

A direct comparison of adsorption properties of our material with other ones reported in the literature is not easy since the adsorption process depends on the adsorbent surface properties, i.e., surface area and the amount and affinity of active sites for nitrate ions, and many experimental parameters, e.g., pH, temperature, adsorbent/adsorbate ratios, etc. For example, a direct comparison of our results with adsorption capacities shown by different powders is challenging since the morphology and porosity of a powder is highly different from that of a compact film, thereby affecting the transfer of contaminants to active sites. Similarly, also in the case of other polymeric adsorbents the comparison is not easy since their surface properties could be totally different. Anyway, we report in Table 7 some results in the literature (considering the same experimental conditions, i.e., neutral pH and room temperature).

Our results are in line with others reported in the literature, even if we have to point out that our results reported in Table 5 could underestimate the adsorption capabilities towards nitrates since we have compared the total mass of the membrane and not the active imidazole sites (i.e., 61 %mol).

## 4. Conclusions

In this work, a pentablock polymeric membrane is proposed for the removal of anionic contaminants, such as nitrate ions and organic dyes (i.e., methyl orange), from wastewater by adsorption. The PTBr membrane is characterized by a copolymer pentablock chain that confers high mechanical, thermal, and chemical stability with the presence of imidazole functionalization balanced by bromide counterions. Chemical characterizations (both IR and XPS) confirm that a washing step removes bromine ions leaving the imidazole active sites free for anions adsorption. Furthermore, the thermal stability of w-PTBr films increases as a consequence of bromine removal and the formation of hydrogen bonds network between imidazole groups on the polymer chains. The w-PTBr film adsorbs nitrate ions with increasing efficiency by increasing the initial nitrate concentration. By kinetic and isotherm analysis it is shown that the adsorption mechanism consists of surface interaction of nitrate ions with imidazole groups (being competitive but predominant with respect to water molecules), followed by diffusion of anions from the solution to the film surface.

PTBr membrane is also able to remove organic contaminants such as MO molecules; these are totally removed within the experimental conditions used in this work and the membrane/dye interaction is governed by surface interactions between the dye and the membrane.

In order to simulate a situation closer to real wastewater, the w-PTBr membrane was tested for the removal of MO and nitrate in mixed solution. Both contaminants were removed, and the kinetic analysis showed competition between the two anions for diffusion from the liquid to the membrane surface and for interaction with active sites. In particular, the adsorption of nitrate was slightly reduced by the presence of MO being competitive for the imidazole active sites, while MO adsorption was quite similar with or without the presence of nitrate ions. This means that, even if direct electrostatic interactions of contaminants with positively charged functional groups on the polymer backbone play the main role in anions removal, other interactions with the membrane (i.e., π-π interactions with the aromatic rings of the polymeric skeleton) can occur, favoring the removal of organic species, such as in the case of MO dye.

In conclusion, the possible use of PTBr for the simultaneous removal of anionic contaminants was demonstrated with minimal impact on the adsorption efficiency of each species.

## Figures and Tables

**Table 1:** The relative contributions (%) calculated by deconvolution of each element peak reported in Figure 3. Dashes indicate that the specific contribution was absent.

Peak	Contributions	PTBr		w-PTBr	
		Peak BE (eV)	(%)	Peak BE (eV)	(%)
C1s	Carbide / C-H / C vacancies	282.00	9.5	282.3	14
	C-C	284	90.5	284.1	75
	C-N	--	--	286.1	11
N1s	R=N-C	--	--	398.3	26.7
	NH (amine)	399.9	24.3	399.7	51.7
	NH_4_^+^	401.3	75.7	401.2	21.6
Br3d	Br 3d_5/2_	67.3	59.8	67.2	59.8
	Br 3d_3/2_	68.3	40.2	68.2	40.2
O1s	O=C-O	530.1	88.6	529.7	16.4
	O=C	531.7	11.4	531.2	45.0
	HO-C	--	--	532.5	38.6

**Table 2:** Q_t_ values measured as mg of adsorbed values for moles of polymeric film or moles of active imidazole sites.

NO_3_^−^ Concentration	Q_t_ (NO_3_^−^ mg/PTBr Moles)	Q_t_ (NO_3_^−^ mg/Imidazole Moles)
1.55	76,260	125,016
3.10	205,820	337,409
7.75	500,200	820,000
15.50	832,300	1,364,426

**Table 3:** Pseudo-first-order (PFO) parameters for nitrate adsorption on PTBr at different initial concentrations.

NO_3_^−^ (mg/L)	R^2^	k_1_ (min^−1^)	Q_e_ (mg/g)	Q_e_^24h^(mg/g)
1.55	0.995	−0.0136 ± 0.0002	1.21	1.12
3.10	0.992	−0.0079 ± 0.0002	2.46	2.51
7.75	0.971	−0.0104 ± 0.0005	6.53	6.10
15.50	0.991	−0.0100 ± 0.0002	9.55	10.50

**Table 4:** Kinetic parameters for the PFO, PSO, and intraparticle diffusion models, respectively.

PFO			PSO			Interparticle Diffusion		
R^2^	k_1_ (min^−1^)	Q_e_ (mg/g)	R^2^	k_2_ (g/mg) min^−1^	Q_e_ (mg/g)	R^2^	K (mg/g) min^0.5^	C
0.992	−0.0035	2.29	0.782	0.0038	2.00	0.978	0.0938	−0.15

**Table 5:** Q_t_ values obtained after 24 h for nitrate ions and MO molecules removal alone or in mixed solution.

Solutions	Q_t_ (mg/g)	
	NO_3_^−^	MO
KNO_3_	10.15	-
MO	-	2.35
KNO_3_ + MO	8.48	2.89

**Table 6:** Kinetic parameters of the PFO, PSO, and intraparticle diffusion models for NO_3_^−^ and MO adsorption in mixed solution. The indicated models provide the best fitting for each contaminant and each time range, as extracted from Table S6.

15.50 mg/L NO_3_^−^								
After 1 h			After 3 h			Up to 330 min		
Intraparticle Diffusion			PFO			PSO		
R^2^	K (mg/g)min^0.5^	C	R^2^	k_2_ (min^−1^)	Q_e_ (mg/g)	R^2^	k_2_ (g/mg)min^−1^	Q_e_ (mg/g)
0.996	0.8645	−2.16	0.998	−0.0015	9.51	0.999	0.0016	9.35
**3.27 mg/L Methyl Orange**								
**After 1 h**			**After 3 h**			**Up to 330 min**		
**Intraparticle Diffusion**			**PFO**			**PFO**		
**R^2^**	**K** **(mg/g)min^0.5^**	**C**	**R^2^**	**k_1_** **(min^−1^)**	**Q_e_** **(mg/g)**	**R^2^**	**k_1_** **(g/mg)min^−1^**	**Q_e_** **(mg/g)**
0.972	0.0751	−0.11	0.993	−0.0013	2.05	0.997	−0.0007	1.84

**Table 7:** Adsorption capacity of some adsorbents for nitrate removal.

Adsorbents		Adsorption Capacity (mg/g)	References
**w-PTBr**		10.15	This work
**Steel slag**		2.83	[68]
**Modified SiO_2_**		20	[69]
**Polystyrene microspheres modified with trimethylamine functional groups**	XAD-4	8	[70]
	GAC	3	
**Bifunctional polystyrene microspheres**	GAC	0	[71]
	XAD-4	3	
**Polyacrylic anion exchange resin**	D311	28	[72]
	AEE-3	24	
**Polymer-based anion exchange fibers**	PAN-PEI-3C	31.30	[73]
	PAN-PEI-5C	31.32	
	PAN-PEI-8C	31.19	

## Data Availability

Dataset available on request from the authors.

## Supplementary Materials

The following supporting information can be downloaded at: https://www.mdpi.com/article/10.3390/polym17121624/s1. Figure S1. SEM image of PTBr film cross section; Figure S2: Survey spectra of PTBr film before and after the washing treatment; Figure S3: C1s (a), O1s (b), N1s (c), and Br3d (d) XPS spectra acquired on PTBr film before and after the washing treatment; Figure S4. UV–Visible spectrum of NO3- solutions within a range of concentrations between 1.55–12,400 mg/L; Figure S5: UV–Vis absorbance spectra over adsorption time for different initial NO3- concentrated solutions in the presence of a w-PTBr membrane: (a) 1.55 mg/L; (b) 3.10 mg/L; (c) 7.75 mg/L, and (d) 15.50 mg/L; Figure S6: Linear fitting of PFO, PSO, and diffusion kinetic models for nitrate adsorption at 1.55, 3.10, 7.75, and 15.50 mg/L initial concentrations up to 330 min; Figure S7. Linear fitting of PFO, PSO, and diffusion kinetic models for nitrate adsorption at 1.55 mg/L initial concentration in three different time regions, i.e., 10–60 min, 60–180 min, and up to 330 min; Figure S8. Linear fitting of PFO, PSO, and diffusion kinetic models for nitrate adsorption at 3.10 mg/L initial concentration in three different time regions, i.e., 10–60 min, 60–180 min, and up to 330 min; Figure S9. Linear fitting of PFO, PSO, and diffusion kinetic models for nitrate adsorption at 7.75 mg/L initial concentration in three different time regions, i.e., 10–60 min, 60–180 min, and up to 330 min; Figure S10. Linear fitting of PFO, PSO, and diffusion kinetic models for nitrate adsorption at 15.50 mg/L initial concentration in three different time regions, i.e., 10–60 min, 60–180 min, and up to 330 min; Figure S11. Freundlich (a) and Langmuir (b) plots for nitrate adsorption on w-PTBr films; Figure S12. Linear fitting of kinetic models for MO adsorption at 3.27 mg/L initial concentrations up to 330 min: (a) PFO; (b) PSO; and (c) diffusion; Figure S13. (a) UV–Visible absorbance spectra of 15.50 mg/L NO3- solution (black curve), of 3.27 mg/L MO solution (orange curve) and of mixed solution at the same concentrations (black curve); (b) a magnification reporting the region between 200 and 300 nm of spectra where nitrate ions peak is clearly visible; Figure S14. Linear fitting of kinetic models for nitrate and MO adsorption in mixed solutions up to 330 min: (a) PFO; (b) PSO; and (c) diffusion; Figure S15. Linear fitting of PFO, PSO, and diffusion kinetic models for MO adsorption at 3.27 mg/L initial concentration in mixed solutions considering three different time regions, i.e., 10–60 min, 60–180 min, and up to 330 min; Figure S16. Linear fitting of PFO, PSO, and diffusion kinetic models for nitrate adsorption at 15.50 mg/L initial concentration in mixed solutions considering three different time regions, i.e., 10–60 min, 60–180 min, and up to 330 min. Table S1: The peak area ratios of N1s, O1s, and Br3d with respect to C1s calculated from XPS spectra before and after the washing treatment; Table S2. Fitting parameters of PFO, PSO, and diffusion kinetic models for nitrate adsorption at 1.55, 3.10, 7.75, and 15.50 mg/L initial concentrations up to 330 min; Table S3. Fitting parameters of PFO, PSO, and diffusion kinetic models for nitrate adsorption at 1.55, 3.10, 7.75, and 15.50 mg/L initial concentration in three different time regions, i.e., 10–60 min, 60–180 min, and up to 330 min; Table S4. Fitting parameters of Langmuir and Freundlich isotherm for nitrate adsorption on w-PTBr films; Table S5. Fitting parameters of all kinetic models for MO adsorption on w-PTBr film; Table S6. Fitting parameters of all kinetic models for nitrate or MO adsorption on w-PTBr film in mixed solutions up to 330 min; Table S7. Fitting parameters of PFO, PSO, and diffusion kinetic models for nitrate at 15.50 mg/L and MO adsorption at 3.27 mg/L concentrations in three different regions, i.e., 10–60 min, 60–180 min, and up to 330 min.
