# Supplementary material for: Kinetic and Isotherm Studies of Organic and Inorganic Anions Adsorption from Water by Quaternized Pentablock Copolymeric Film (PTBr)
Source: Polymers (Basel). 2025 Jun 11;17(12):1624. doi: 10.3390/polym17121624 (PMC12196666; doi:10.3390/polym17121624)
Supplement: Supplementary file 1 [file polymers-17-01624-s001.zip › polymers-3662274-supplementary.pdf]

Kinetic and isotherm studies of organic and inorganic anions adsorption from water by quaternized pentablock copolymeric film (PTBr)

Simona Crispi[1], Simona Filice[1], Viviana Scuderi[1], Massimo Zimbone[2], Daniela Iannazzo[3], Consuelo Celesti[3] and Silvia Scalese[1]*

[1]Consiglio Nazionale delle Ricerche, Istituto per la Microelettronica e Microsistemi (CNR-IMM), Ottava Strada n.5, 95121 Catania, Italy: simona.crispi@cnr.it, viviana.scuderi@imm.cnr.it, silvia.scalese@imm.cnr.it;

[2]Consiglio Nazionale delle Ricerche, Istituto per la Microelettronica e Microsistemi (CNR-IMM), Via S. Sofia 64, 95123 Catania, Italy: massimo.zimbone@cnr.it;

[3] Department of Engineering, University of Messina, Contrada Di Dio, I-98166 Messina, Italy: daniela.iannazzo@unime.it; consuelo.celesti@unime.it;

*Correspondence: simona.filice@cnr.it.

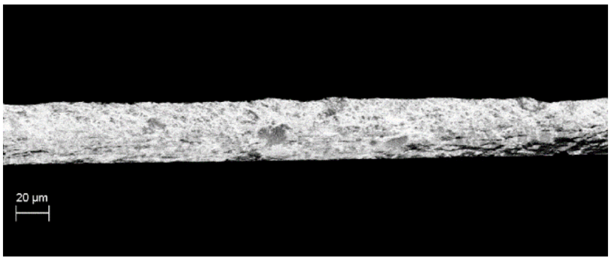

Figure S1. SEM image of PTBr film cross section.

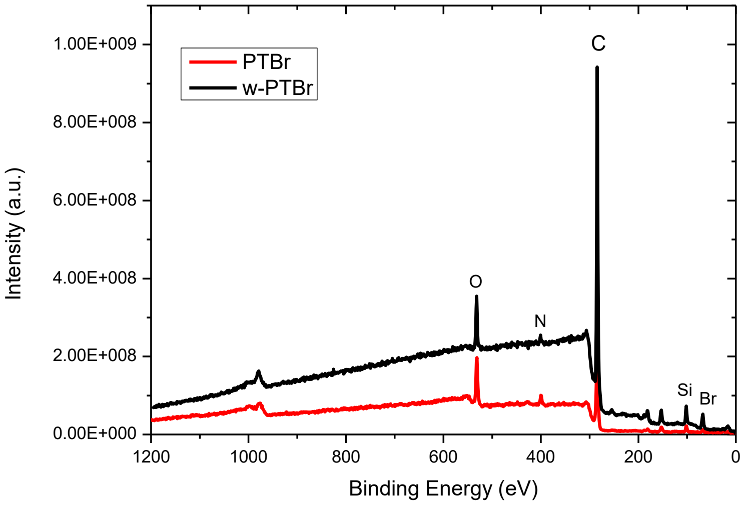

Figure S2. Survey spectra of PTBr film before and after the washing treatment.

Table S1. The peak area ratios of N1s, O1s and Br3d with respect to C1s calculated from XPS spectra before and after the washing treatment.

Element ratio	PTBr	w-PTBr
N/C	0.146	0.094
O/C	0.759	0.455
Br/C	0.205	0.0195

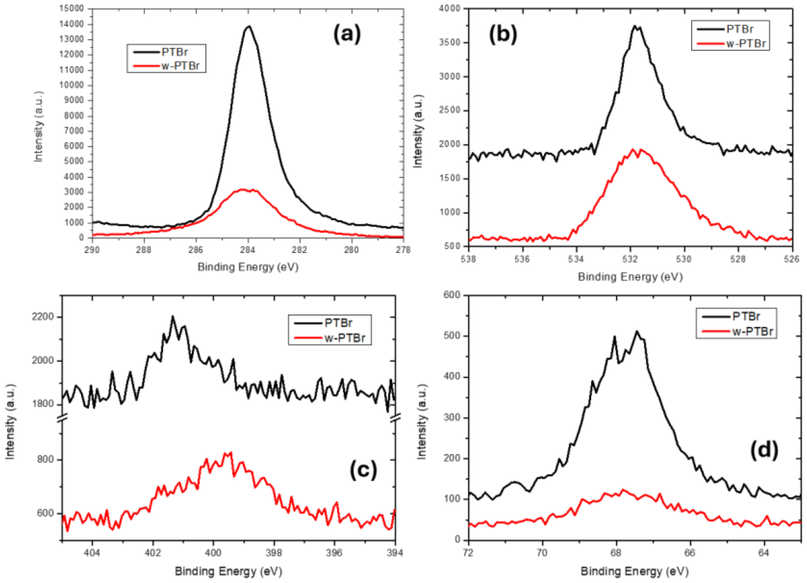

Figure S3. C1s (a), O1s (b), N1s (c) and Br3d (d) XPS spectra acquired on PTBr film before and after the washing treatment.

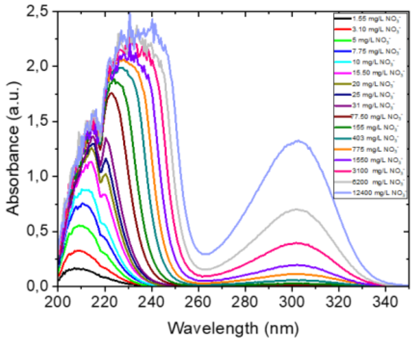

Figure S4. UV-Visible spectrum of NO_3^- solutions within a range of concentrations between 1.55 - 12400 mg/L.

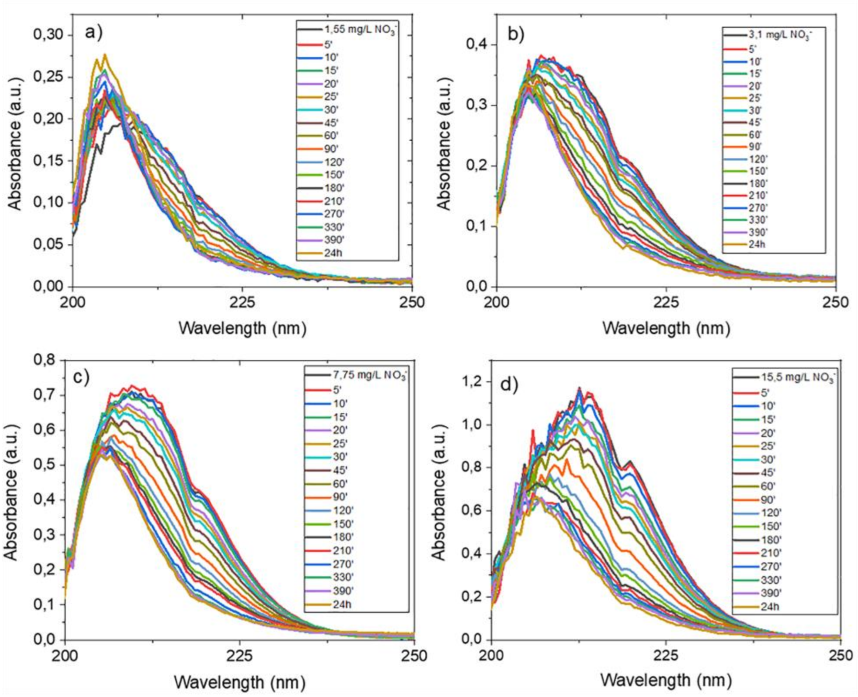

Figure S5. UV-Vis absorbance spectra over adsorption time for different initial NO_3^- concentrated solutions in the presence of a w-PTBr membrane: (a) 1.55 mg/L; (b) 3.10 mg/L; (c) 7.75 mg/L and (d) 15.50 mg/L.

Table S2. Fitting parameters of PFO, PSO and diffusion kinetic models for nitrate adsorption at 1.55, 3.10, 7.75 and 15.50 mg/L initial concentrations up to 330 minutes.

**Nitrate initial concentration (mg/L)**	**Kinetic model**	**R^2**	**SLOPE**	**INTERCEPT**
1.55	PFO	0.995	-0.0136 ± 0,0002	0.1870 ± 0,0351
	PSO	0.014	0.9915 ± 0.8977	2.1257 ± 125.2019
	DIFF	0.940	0.0770 ± 0.0050	-0.0967 ± 0.0495
3.10	PFO	0.992	-0.0079 ± 0.0002	0.9018 ± 0.0251
	PSO	0.065	0.1983 ± 0.1388	62.3801 ± 19.3546
	DIFF	0.983	0.1481 ± 0.0050	-0.1767 ± 0.0495
7.75	PFO	0.971	-0.0104 ± 0.0005	1.8776 ± 0.0648
	PSO	0.394	0.1327 ± 0.0404	13.0249 ± 5.6389
	DIFF	0.968	0.3884 ± 0.0181	-0.5354 ± 0.1789
15.50	PFO	0.991	-0.0100 ± 0.0002	2.2561 ± 0.0336
	PSO	0.015	0.0433 ± 0.0390	14.3544 ± 5.4415
	DIFF	0.955	0.6412 ± 0.0358	-0.4278 ± 0.3536

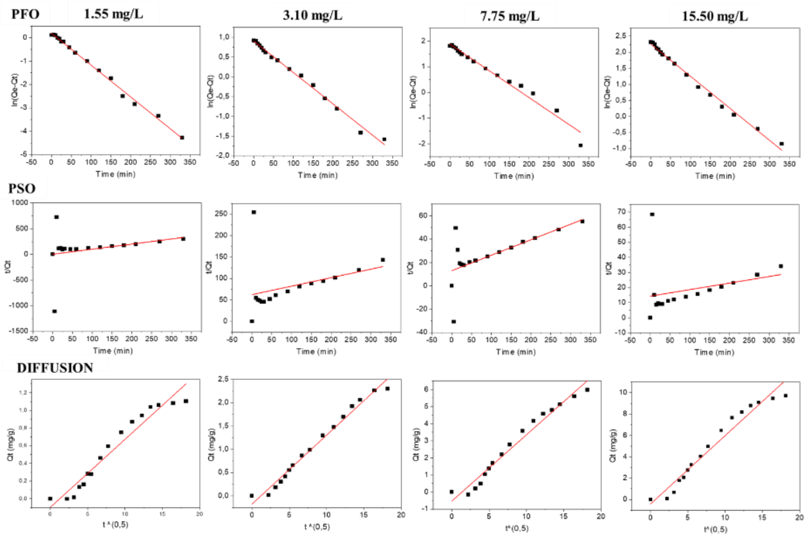

Figure S6. Linear fitting of PFO, PSO and diffusion kinetic models for nitrate adsorption at 1.55, 3.10, 7.75 and 15.50 mg/L initial concentrations up to 330 minutes.

Table S3. Fitting parameters of PFO, PSO and diffusion kinetic models for nitrate adsorption at 1.55, 3.10, 7.75 and 15.50 mg/L initial concentration in three different time regions i.e. 10-60 minutes, 60-180 minutes and up to 330 minutes.

**Nitrate concentrations (mg/L)**	**Kinetic Models**	**Process Time**								
		**10-60 min**			**60-180 min**			**180-330 min**		
		**R^2**	**SLOPE**	**INTERCEPT**	**R^2**	**SLOPE**	**INTERCEPT**	**R^2**	**SLOPE**	**INTERCEPT**
1.55	PFO	0.989	-0.0145 ± 0.0007	0.2272 ± 0.0219	0.960	-0.0148 ± 0.0015	0.3204 ± 0.1905	0.971	-0.0114 ± 0.0011	-0.4085 ± 0.2892
	PSO	0.1011	-6.6125 ± 5.1097	387.4733 ± 171.3840	0.997	0.6150 ± 0.0182	64.4688 ± 2.3208	0.999	0.8373 ± 0.0050	22.8381 ± 1.273
	DIFF	0.986	0.1237 ± 0.0061	-0.3701 ± 0.0330	0.991	0.0767 ± 0.0037	0.0126 ± 0.0399	0.972	0.0138 ± 0.0013	0.8549 ± 0.0210
3.10	PFO	0.970	-0.0087 ± 0.0006	0.9064 ± 0.0208	0.979	-0.0077 ± 0.0006	0.9083 ± 0.0717	0.915	-0.0072 ± 0.0013	0.6932 ± 0.3175

	PSO	0.148	0.1668 ± 0.1168	46.0193 ± 3.9176	0.974	0.2805 ± 0.0227	44.9729 ± 2.8935	0.988	0.3303 ± 0.0208	32.7753 ± 5.2814
	DIFF	0.987	0.1823 ± 0.0085	-0.3830 ± 0.0461	0.994	0.1615± 0.0062	-0.2619 ± 0.0681	0.892	0.0808 ± 0.0159	0.8754 ± 0.2508
7.75	PFO	0.978	-0.0114 ± 0.007	1.8640 ± 0.0230	0.989	-0.0079 ± 0.0004	1.6526 ± 0.0531	0.944	-0.0153 ± 0.0021	3.1345 ± 0.5413
	PSO	0.144	-0.3479 ± 0.2454	35.5441 ± 8.2294	0.996	0.1307 ± 0.0042	13.5008 ± 0.5358	0.999	0.0108	0.1191 ± 0.0006
	DIFF	0.986	0.5688 ± 0.0281	-1.5667 ± 0.1519	0.976	0.3635 ± 0.0285	0.0624 ± 0.3123	0.992	0.2436 ± 0.0124	1.5736 ± 0.1944
15.50	PFO	0.973	-0.0115 ± 0.0008	2.3142 ± 0.0262	0.995	-0.0110 ± 0.0004	2.2897 ± 0.0509	0.999	-0.0077 ± 0.0009	1.6773 ± 0.0237
	PSO	-0.198	0.0041 ± 0.0596	10.5685 ± 2.0001	0.993	0.0708 ± 0.0030	7.5841 ± 0.3862	0.999	0.0899 ± 0.0003	4.2497 ± 0.0748
	DIFF	0.979	0.9037 ± 0.0531	-1.9129 ± 0.2871	0.971	0.6702 ± 0.0574	-0.0014 ± 0.6286	0.979	0.1932 ± 0.0163	6.2475 ± 0.2561

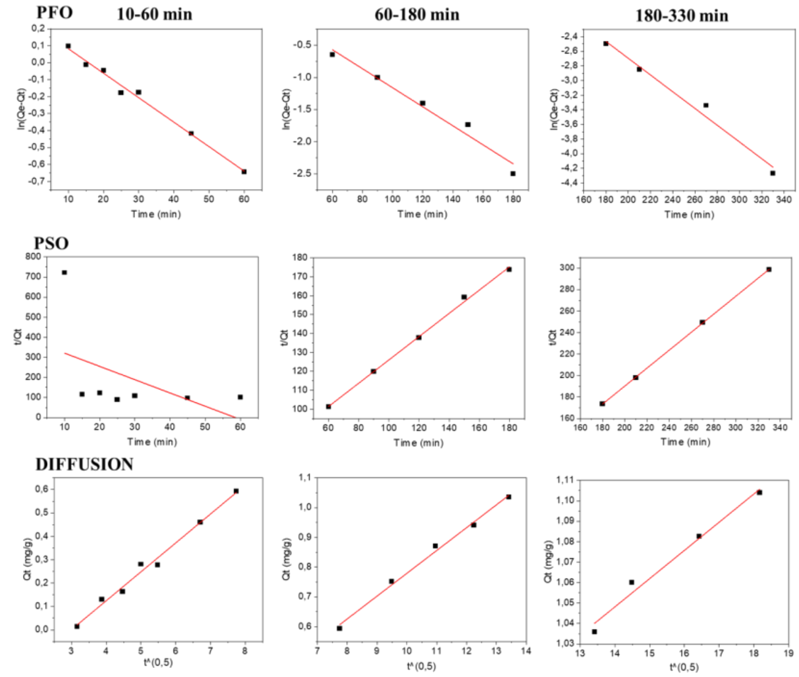

Figure S7. Linear fitting of PFO, PSO and diffusion kinetic models for nitrate adsorption at 1.55 mg/L initial concentration in three different time regions i.e. 10-60 minutes, 60-180 minutes and up to 330 minutes.

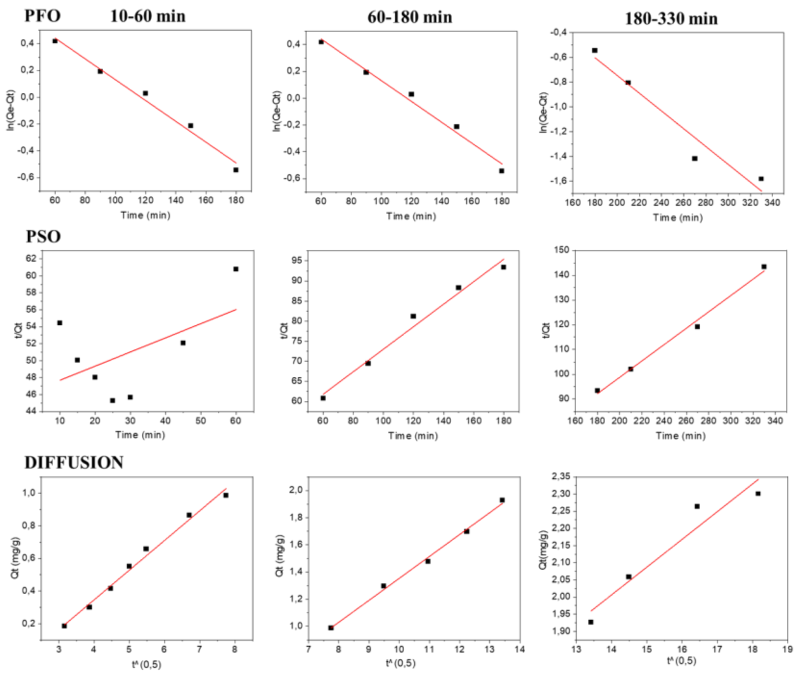

Figure S8. Linear fitting of PFO, PSO and diffusion kinetic models for nitrate adsorption at 3.10 mg/L initial concentration in three different time regions i.e. 10-60 minutes, 60-180 minutes and up to 330 minutes.

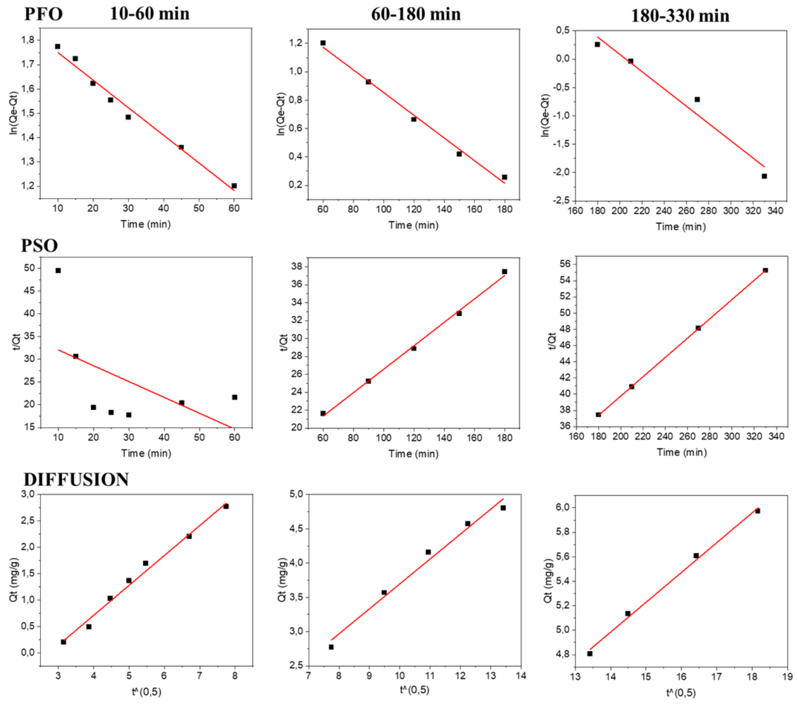

Figure S9. Linear fitting of PFO, PSO and diffusion kinetic models for nitrate adsorption at 7.75 mg/L initial concentration in three different time regions i.e. 10-60 minutes, 60-180 minutes and up to 330 minutes.

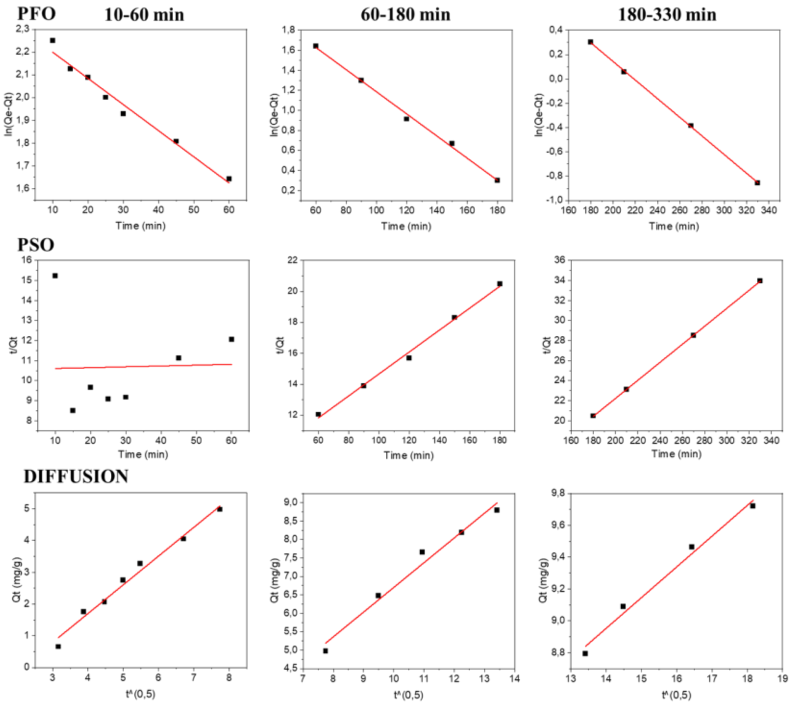

Figure S10. Linear fitting of PFO, PSO and diffusion kinetic models for nitrate adsorption at 15.50 mg/L initial concentration in three different time regions i.e. 10-60 minutes, 60-180 minutes and up to 330 minutes.

Table S4. Fitting parameters of Langmuir and Freundlich isotherm for nitrate adsorption on w-PTBr films.

Langmuir			Freundlich		
Q_{max} (mg/g)	K_L (L/mg)	R^2	1/n	K_F ($L^{1/n}$ $mg^{(1-1/n)}$/g)	R^2
-16.84	18.86	0.997	1.17	2.40	0.994

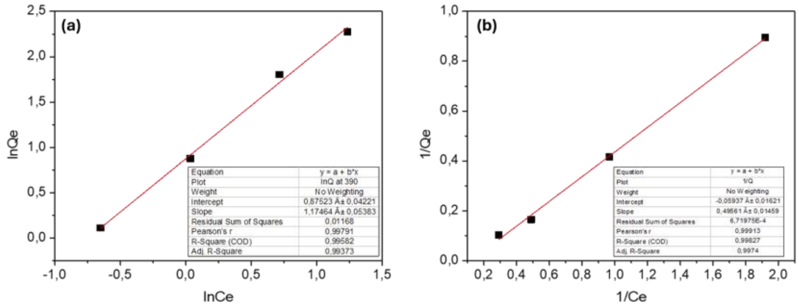

Figure S11. Freundlich (a) and Langmuir (b) plots for nitrate adsorption on w-PTBr films.

Table S5. Fitting parameters of all kinetic models for MO adsorption on w-PTBr film.

MO concentration (mg/L)	Kinetic Models	Fitting Parameters		
		R^2	SLOPE	INTERCEPT
3.27	PFO	0.992	-0.0035 ± 0.0001	0.8282 ± 0.0128
	PSO	0.782	0.4980 ± 0.0749	64.7965 ± 10.4891
	DIFF	0.977	0.0938 ± 0.0041	-0.1447 ± 0.0408

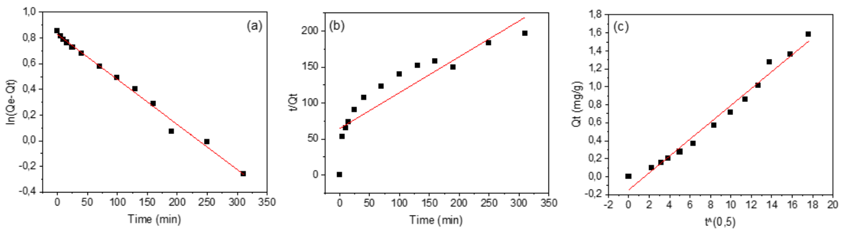

Figure S12. Linear fitting of kinetic models for MO adsorption at 3.27 mg/L initial concentrations up to 330 minutes: (a) PFO; (b) PSO and (c) diffusion.

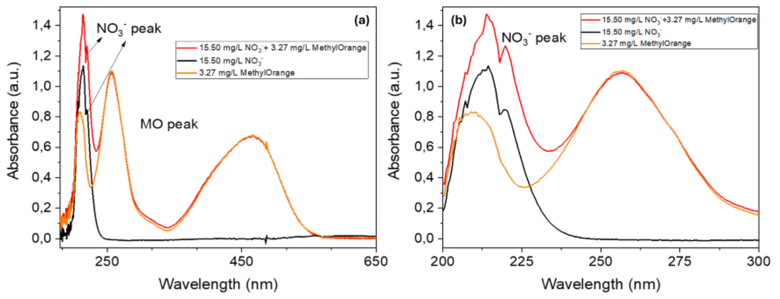

Figure S13. (a) UV-Visible absorbance spectra of 15.50 mg/L NO_3^- solution (black curve), of 3.27 mg/L MO solution (orange curve) and of mixed solution at the same concentrations (black curve). (b) a magnification reporting the region between 200 and 300 nm of spectra where nitrate ions peak is clearly visible.

Table S6. Fitting parameters of all kinetic models for nitrate or MO adsorption on w-PTBr film in mixed solutions up to 330 minutes.

Contaminants	Kinetic Model	Fitting Parameters		
		R^2	SLOPE	INTERCEPT
MO in mixed solution	PFO	0.909	-0.0014 ± 0.0001	0.7722 ± 0.0157
	PSO	0.958	1.0082 ± 0.0542	63.1430 ± 7.554
	DIFF	0.983	0.0511 ± 0.0017	0.0103 ± 0.0169
KNO_3 in mixed solution	PFO	0.925	-0.0048 ± 0.0003	2.1755 ± 0.0485
	PSO	-0.037	0.0426 ± 0.0629	20.4611 ± 8.7604
	DIFF	0.950	0.5064 ± 0.0299	-0.2821 ± 0.2954

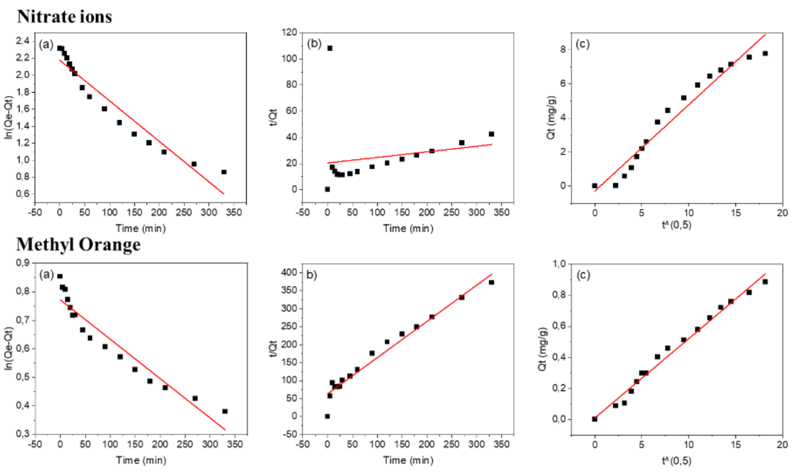

Figure S14. Linear fitting of kinetic models for nitrate and MO adsorption in mixed solutions up to 330 minutes: a) PFO; b) PSO and c) diffusion.

Table S7. Fitting parameters of PFO, PSO and diffusion kinetic models for nitrate at 15.50 mg/L and MO adsorption at 3.27 mg/L concentrations in three different regions i.e. 10-60 minutes, 60-180 minutes and up to 330 minutes.

**Solution**	**Kinetic Models**	**10-60**			**60-180**			**180-330**		
		**R^2**	**SLOPE**	**INTERCEPT**	**R^2**	**SLOPE**	**INTERCEPT**	**R^2**	**SLOPE**	**INTERCEPT**
MO in mixed solution	PFO	0.933	-0.0032 ± 0.0004	0.8186 ± 0.0119	0.993	-0.0013 ± 0.0001	0.7184 ± 0.0069	0.997	-0.0007± 0.0001	0.6118 ± 0.0057
	PSO	0.768	0.9291 ± 0.2035	70.6963 ± 6.826	0.959	0.9679 ± 0.0989	82.3249 ± 12.5878	0.994	0.8261 ± 0.0361	102.7447 ± 9.1794
	DIFF	0.972	0.0751 ± 0.0052	-0.1066 ± 0.0283	0.977	0.0570 ± 0.0036	0.0793 ± 0.0395	0.994	0.0339 ± 0.0015	0.2656 ± 0.0231
KNO_3 in mixed solution	PFO	0.989	-0.0104 ± 0.0004	2.3466 ± 0.0151	0.992	-0.0046 ± 0.0002	2.0109 ± 0.0260	0.961	-0.0023 ± 0.0003	1.5860 ± 0.0664
	PSO	-0.072	-0.0378 ± 0.0489	14.1061 ± 1.6411	0.998	0.1052 ± 0.0030	7.5451 ± 0.3841	0.999	0.1069 ± 0.0016	7.0271 ± 0.4165
	DIFF	0.996	0.8640± 0.0232	-2.1617 ± 0.1254	0.993	0.4315 ± 0.0181	1.1124 ± 0.1979	0.958	0.2011 ± 0.0241	4.1833 ± 0.3788

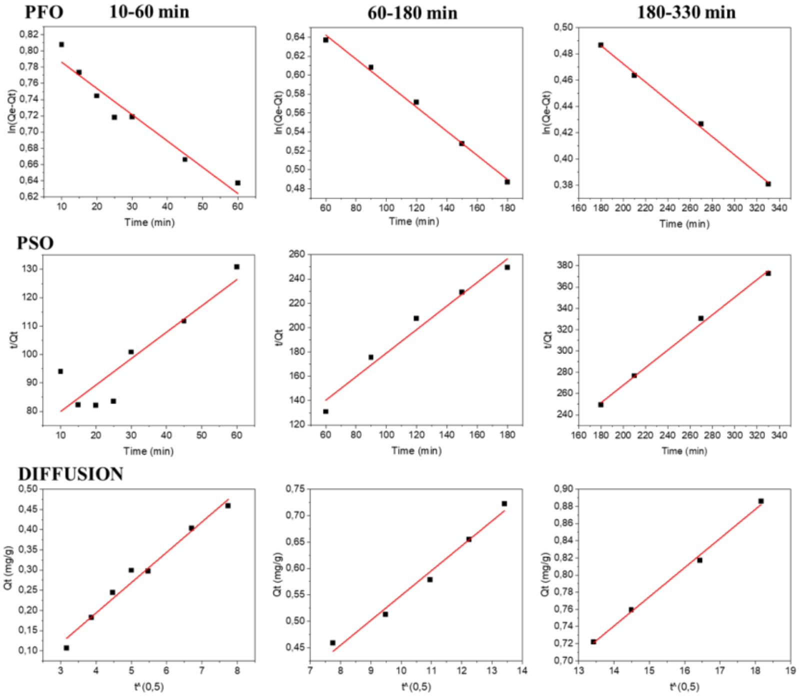

Figure S15. Linear fitting of PFO, PSO and diffusion kinetic models for MO adsorption at 3.27 mg/L initial concentration in mixed solutions considering three different time regions i.e. 10-60 minutes, 60-180 minutes and up to 330 minutes.

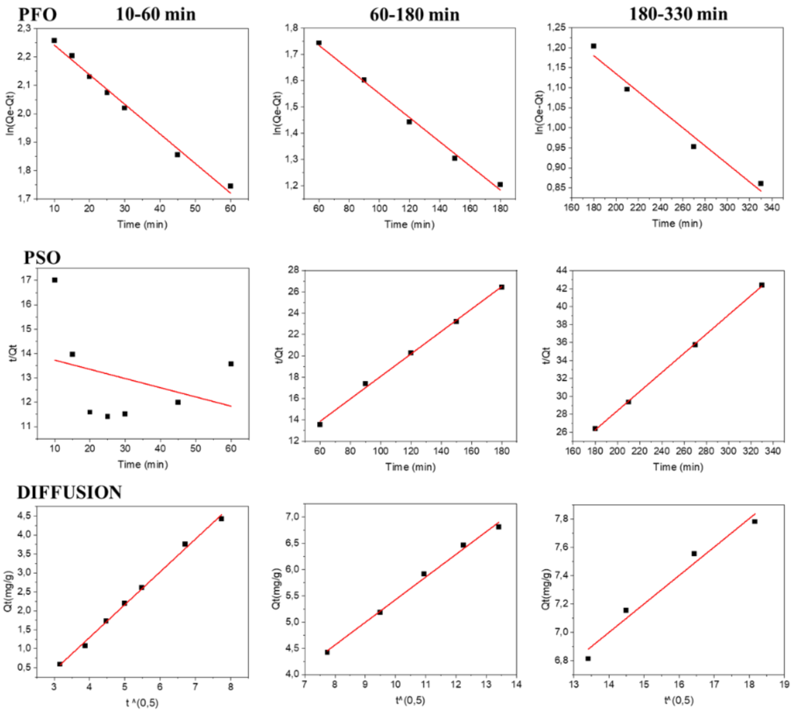

Figure S16. Linear fitting of PFO, PSO and diffusion kinetic models for nitrate adsorption at 15.50 mg/L initial concentration in mixed solutions considering three different time regions i.e. 10-60 minutes, 60-180 minutes and up to 330 minutes.
